# Cardioprotective potential of the antioxidant-rich bioactive fraction of *Garcinia pedunculata* Roxb. ex Buch.-Ham. against isoproterenol-induced myocardial infarction in Wistar rats

**DOI:** 10.3389/fphar.2022.1009023

**Published:** 2022-10-04

**Authors:** Swarnali Bhattacharjee, R. Elancheran, Kasturi Dutta, Prashanta Kumar Deb, Rajlakshmi Devi

**Affiliations:** ^1^ Life Sciences Division, Institute of Advanced Study in Science and Technology, Guwahati, Assam, India; ^2^ Department of Zoology, Gauhati University, Guwahati, Assam, India; ^3^ Department of Chemistry, Annamalai University, Chidambaram, TamilNadu, India; ^4^ Department of Pharmaceutical Chemistry, School of Pharmaceutical Sciences, Shoolini University, Solan, Himachal Pradesh, India

**Keywords:** cardioprotective effect, isoproterenol-induced myocardial infarction, Garcinia pedunculata, phytocompounds, molecular modelling

## Abstract

This Study aimed to characterise the phenolic compounds in *Garcinia pedunculata* extract and assess their potential antioxidant activity as well as its cardioprotective potential in isoproterenol-induced cardiac hypertrophy in an experimental animal model. *In vitro* antioxidant properties were determined using DPPH, ABTS, FRAP, PMD assays. *In vitro* lipid peroxidation experiment was also performed with heart tissues. Cardioprotective and cardiotoxicity effects were determined using the cell line studies. The cardioprotective effect of GP was assessed in a rat model of isoproterenol-(ISO-) induced cardiac hypertrophy by subcutaneous administration. Heart weight/tail length ratio and cardiac hypertrophy indicators were reduced after oral administration of GP. Additionally, GP reduced oxidative stress and heart inflammation brought on by ISO. In H9c2 cells, the antihypertrophic and anti-inflammatory effects of the extract of GP were seen in the presence of ISO, which were further supported by the *in vivo* observations. This study makes a compelling case for the possibility that supplementing with dried GP fruit can prevent heart hypertrophy by reducing oxidative stress and inflammation.

## 1 Introduction

The World Health Organisation (WHO) commissioned the Global Burden of Disease (GBD) study in the 1990s, where childhood illness, psychological diseases, and traffic accidents were the leading causes of death worldwide (Mathers, 2020). By the statistics of 2019, the burden shifted to non-communicable diseases (NCDs), claiming about 42 million lives globally, and cardiovascular diseases (CVDs) are among its leading cause independent of the socioeconomic background of the countries (Emadi-Emadi et al., 2021). About 1/3rd of the global deaths happen due to cardiovascular diseases, the most rampant of them being ischemic heart disease (IHD). Myocardial infarction is the most common manifestation of IHD, which might come in the form of coronary artery disease and atherosclerosis (Khan et al., 2020). In the Indian scenario, NCDs were also on the rise from the 1990s to 2016, with CVDs being the principal cause of annual mortality (Mohan et al., 2019). Myocardial infarction (MI), in general terms, heart attack, happens with obstructed circulation leading to localized necrosis in the myocardium with pronounced ischemia. The most common diagnostic criteria include an increase in cardiac troponin I or T and ST-wave elevation or depression in ECG analysis (Saleh & Ambrose, 2018). Initial markers for MI were aspartate transaminase in the mid-1990s, which was then replaced with more specific markers like creatinine kinase (CK), CK-MB, and lactate dehydrogenase (LDH). A widely accepted gold-standard marker for MI was found in elevated troponin T or I levels. The other biomarkers, although not diagnostic, include B-Type natriuretic peptide (BNP), C reactive protein (CRP), IL-6, and TNF-α (Chen et al., 2019a). The patients hospitalised with chest pain are often suspected of MI, and are diagnosed based on advance techniques like electrocardiography (ECG); echocardiography, coronary angiography, cardiac MRI and CT-scan along with the biomarkers (Sandoval & Jaffe, 2019). A multitude of risk factors are associated with MI, of which some prominent modifiable risk factors include smoking, alcohol abuse, diet rich in fats and carbohydrates, lacking fruits and vegetables, sedentary lifestyle, unhealthy weight gain, hypertension, and hyperlipidemia (Ojha and Dhamoon, 2021).

An ideal model to mimic the human MI-related complications is the isoproterenol-induced myocardial injury non-invasive model used to study natural products’ cardioprotective or preventive potential. Isoproterenol is a synthetic catecholamine, ß- receptor agonist. A 85 mg/kg of ISO dosage induces myocardial necrosis, degeneration of myofibrils, fibrosis, inflammation, calcium overload, and energy deficit in the rat heart (Wong et al., 2017).

With the impending side effects from the prolonged use of synthetic medicines for cardiac complications, there is an urgent need for safer alternatives. With this in mind, focused research on natural products of herbal origin with negligible side effects to explore their cardioprotective potential came into being (Allawadhi et al., 2018; Shah et al., 2019). Over the years, several plants like *Amaranthus viridis*, *Ginkgo biloba*, *Terminalia arjuna*, *Tinospora cordifolia*, *Curcuma longa*, *Hydrocotyle asiatica*, *Centella asiatica*, *Allium sativum*, *Withania somnifera*, *Garcinia kola, Zingiber officinale,* and many others have been assessed for their cardioprotective potential (Shah et al., 2019; Kumar et al., 2017). Some of the plant-derived bioactive compounds with potent cardioprotective activity include digoxin (arrhythmia treatment), atropine (bradycardia treatment), allicin, kolaviron, caffeic acid, epigallocatechin-3-gallate, resveratrol, carotenoids, with their therapeutic activities like potent antioxidant anti-hypertensive, anti-atherosclerotic, anti-inflammatory, and improving insulin sensitivity properties to name a few (Shah et al., 2019; Sharifi-Rad et al., 2020). One such fruit of interest is *Garcinia pedunculata* Roxb. or GP.

*G. pedunculata*, an evergreen tree belonging to the Clusiaceae family, bears an acidic, globose fruit with immense therapeutic potential and is a well-known medicinal plant belonging to Northeast India. Although the consumption of raw fruit is traditionally forbidden, the ripened fruit is used to treat gastrointestinal disorders and is lavishly used in cooking various delicacies of Assamese cuisine. Among the prominent medicinal properties reported from the fruit of GP include antioxidant, hypolipidemic, nephroprotective, neuroprotective, and anti-diabetic properties. Also, chemical profiling of GP unraveled multiple compounds like hydroxycitric acid, 2,4,6,3′,5′-pentahydroxybenzophenone, pedunculol, and pedunxanthone A from different parts of the plant (Bhattacharjee and Devi, 2021). Despite the already established therapeutic properties, reports on GP fruit’s cardioprotective potential are minimal. Keeping this research gap in mind, this study has been designed to assess the cardioprotective potential of the polyphenol-rich fraction of GP fruit using isoproterenol-induced MI in Wistar rats along with its chemical profiling.

## 2 Materials and methods

### 2.1 Chemicals

Chloroform, Ethyl acetate, Hexane, Methanol (analytical grade, Merck); Sodium carbonate, Folin Ciocalteu reagent, Gallic acid, 1, 1-Diphenyl-2-picrylhydrazyl (DPPH), 2,2′-Azino-bis(3-ethylbenzthiazoline-6-sulfonic acid (ABTS), Potassium ferricyanide, Disodium hydrogen phosphate, Monosodium phosphate, Trichloroacetic acid, Iron (III) chloride, Iron (II) sulphate, MTT (3-(4,5-Dimethylthiazol-2-yl)-2,5-Diphenyltetrazolium Bromide), 2′,7′-dichlorodihydrofluorescein diacetate (H2DCFDA), Thiobarbituric acid, Glacial acetic acid, Trolox, Sodium dodecyl phosphate, Pyrogallol, Ellman’s reagent, hydrogen peroxide were purchased from Sigma-Aldrich, United States and Merck, Germany.

### 2.2 Plant materials

GP fruit was collected from the Kamrup district (25°43՛ - 26°19՛ North latitude and 90°39՛- 91°47՛ East latitude), Guwahati, Assam and was taxonomically verified from the Department of Botany, Gauhati University under voucher number Ref. No. *Herb./GUBH/2022/002* and have been submitted to Gauhati University, Herbarium section, under accession number GUBH19815.

### 2.3 Plant material processing, extraction, and fractionation

The fruits were thoroughly cleaned, and their pulp and rinds were thinly sliced and dried in the Sun without the seeds. Maceration was used to prepare the crude extract (GPC) of the fruit in methanol, and the dried crude extract was then fractionated using column fractionation (Silica, 60–120 mesh) with the appropriate solvents: hexane (GH), chloroform (GC), ethyl acetate (GE), and methanol (GM). A rotary evaporator (Buchi R210, Switzerland) was used to remove the solvents from the extracts, and the material was subsequently lyophilized (FreeZone Freeze Dryer, Labconco). The yield percentage for each extract was calculated, and until usage, it was kept in sealed containers at 4°C–8°C.

### 2.4 Total phenolic content

The phenolic content was estimated using the methodology of Sarma et al., 2016 with slight modification by adding 1,000 μL of 2% Sodium carbonate (freshly prepared), 100 μL of Folin-Ciocalteu reagent to 100 μL of different extracts and fractions at a concentration of 10 mg/ml, which were mixed well and left in the dark for 30 min of incubation. The absorbance of the resultant mixture was measured spectrophotometrically in a microplate reader (Multiskan GO, Thermo, Germany) at 750 nm. The standard curve was prepared with Gallic acid (y = 0.006x+ 0.322, R^2^ = 0.988), and the data were expressed as mg GAE/g (Gallic acid equivalent).

### 2.5 UHPLC-ESI orbitrap MS/MS analysis

An ultra-high pressure liquid chromatography (UHPLC) system from Dionex Inc. In Sunnyvale, California, was used to perform the chromatographic separation. This system includes a binary pump, a degasser, an autosampler, a thermostated column compartment, and a control module. Chromatographic separation was performed using a UHPLC system connected to an Electron Spray Ionization (ESI) Orbitrap mass spectrometer, and Hypersil Gold C18 column operating at 25 °C. Using the earlier technique, gradient chromatographic separation was carried out for each extract of the samples (Kumari et al., 2017). Using the mass spectrum and its unique fragmentation spectra, the phenolic compounds in the *Garcinia* extract were screened and identified. The key method for potential identification of the phenolic compounds was the comparison of the obtained MS/MS spectra with those published in the literature and mass bank database.

### 2.6 *In- vitro* screening of antioxidant potential

#### 2.6.1 1, 1-diphenyl-2-picrylhydrazyl radical scavenging assay

To 100 µL of freshly prepared 0.2 mM DPPH solution, 100 µL of extracts and fractions (prepared in methanol) were added in different concentrations, and the absorbance was recorded at 517 nm after an incubation of 30 min in the dark at room temperature. The resulting IC_50_ for each extract was evaluated to compare the radical scavenging activity. If lower the IC_50_, better the scavenging activity (Iloki-Assanga et al., 2015).

#### 2.6.2 2,2՛-Azinobis (3-ethylbenzthiazoline -6- sulphonic acid (ABTS) radical scavenging assay

The antioxidant activity of the extracts was assessed by the increased ABTS^+^ radical cation scavenging ability with slight modification (Re et al., 1999; Sarma et al., 2016), by adding a 7 mM solution of ABTS in a 1:1 ratio with various quantities of extracts, incubating in the dark for half an hour at room temperature, and measuring absorbance at 734 nm. Following the DPPH experiment, the antioxidant activity was compared.

#### 2.6.3 Ferric reducing assay power

Add 250 μL of 0.2 M phosphate buffer (pH 6.6) and 250 μL of potassium ferricyanide (1%) to 100 μL of extracts and fractions, each taken in a different concentration. After that, this mixture is allowed to stand at 50 °C for 30 min 250 μL of 10% trichloroacetic acid was added to the mixture after incubation. In addition, 250 μL of the resulting mixture was diluted 1:1 with distilled water before being added to a freshly made 0.1 percent ferric chloride solution, which was used to measure the absorbance at 700 nm. Increased antioxidant activity was related to the increase in absorption (Vijayalakshmi & Ruckmani, 2016).

#### 2.6.4 Phosphomolybdenum assay

The 28 mM sodium phosphate and 4 mM ammonium molybdate solution were mixed with 0.6 M sulfuric acid to form the phosphomolybdate reagent, which was then freshly prepared. The fractions and varying concentrations of the extract are mixed with 150 ml of this reagent and incubated for 90 min at 95 °C. The reducing activity is indicated by the increase in absorbance, which was measured at 695 nm (Khan et al., 2012).

#### 2.6.5 *In- vitro* lipid peroxidation assay

Heart tissue homogenate was tested in an *in vitro* lipid peroxidation experiment, where the Fe^2+^ Ascorbate system was used to induce lipid peroxidation. This assay was carried out with some small alterations to (Sarma et al., 2016). 50 mM phosphate buffer (pH 7.4) was used to prepare 10 percent (w/v) tissue homogenate, which was then employed in the test without centrifugation. The following mixtures are added to 250 μL of tissue extract, 100 μL of 150 mM Tris HCL buffer (pH 7.2), 50 μL of 0.1 mM Ascorbic acid, 50 μL of 4 mM FeSO_4_, 50 μL of the extract and fractions in different concentrations, 50 μL of H_2_O, and 50 μL of Trolox as standard. Following that, this mixture is incubated for 1 hour at 37 °C. The following step is Thiobarbituric Acid Reactive Substance Measurement (TBARS), which involves mixing 50 μL of the abovementioned mixture with 150 μL each of 0.8 percent (w/v) thiobarbituric acid, 20 μL of 8.1 percent SDS, and 30 μL of deionized water. This mixture is incubated at 95°C for an hour, cooled under tap water, and then 500 μL of butanol: pyridine (15:1, v/v) and 100 μL of distilled water are added. The upper layer is then carefully removed to measure the absorbance at 532 nm after the mixture has been vortexed and centrifuged at 4,000 rpm for 10 min. The extract with a lower IC_50_ was more effective at inhibiting lipid peroxidation.

### 2.7 *In- vitro* screening of cardioprotective effect in cardiomyocytes

The H9c2 cell line (Rat cardiomyoblast) was procured from NCCS in Pune for use in cell culture experiments. They were subcultured at around 80% confluency in a humidified 5 percent CO_2_ incubator with DMEM (4.5 g/L glucose, Gibco) containing 10% FBS, 1% antibiotic solution, and 37°C. For all the experiments were performed using 2 to 10 passages.

#### 2.7.1 Establishment of isoproterenol induced *in vitro* cardiotoxicity model

H9c2 cells were plated in the required concentrations in 10 percent FBS-containing DMEM for the ISO-treated cardiotoxicity model, and the medium was changed to 1 percent FBS-containing media after 24 h. The cells were treated with various doses of ISO or ISO + extracts after being serum-deprived for 48 h to promote cellular differentiation (Branco et al., 2011; Sivasangari et al., 2019; Raut et al., 2020).

#### 2.7.1 Cell-culture treatment

Different GC concentrations were applied to the cells for the cell culture experiments 2 hours before the 25 µM ISO treatment. Every day, the used media were replaced. The experimental dilutions were prepared in DMEM with the DMSO concentration kept below 0.01 percent, and 0.01 percent was utilised as the vehicle control, while ISO was prepared in PBS. The stock for GC was prepared in DMSO and maintained at -20 °C (pH 7.4, Gibco).

##### 2.7.1.1 Cell viability assay

For the cardiotoxicity model, the cells were seeded at a concentration of 1 × 10^4^ cells/well in 96 well cell culture plate (Tarsons) and treated with different concentrations of isoproterenol, GC, and ISO+GC. Before adding ISO, cells were pretreated with GC extract for 2 hours. The MTT (3-(4,5-dimethylthiazol-2-yl)-2,5-diphenyl tetrazolium bromide) assay was used to determine the viability of the cells, and the absorbance was measured at 570 nm. The viability is determined by comparing the proportion of living cells in treated concentrations to untreated control cells (Sivasangari et al., 2019).

##### 2.7.1.2 Study of cell morphology with ISO treatment

This assay was performed as per the protocol of Sivasangari et al., 2019, and the unstained cells were photographed using a phase-contrast inverted microscope Leica DMI 3000 B, with a scale of 200 µm.

##### 2.7.1.3 LDH release assay

The LDH release assay was used to assess the extent of cellular damage caused by ISO treatment in H9c2 cells, as well as the role of GC in conferring cardioprotection against ISO. The LDH Test Kit (Himedia, CAT: CCK058-500) was used to perform the assay, and the manufacturer’s protocols were followed. The cells were seeded at the same concentration as in the MTT.

##### 2.7.1.4 Intracellular ROS detection

After treatment for 12 h, the spent media was discarded, and the cells were washed with 1X PBS (cell culture grade, pH 7.4, Gibco) twice and then incubated with 10 µM of H_2_DCF-DA in phenol red-free DMEM for 45 min at 37 °C. After incubation, the remaining H_2_DCF-DA were removed and cells were again washed properly with 1X PBS then their fluorescence intensity was measured at 485 nm (excitation) and 525 nm (emission) using a VarioskanFlash microplate reader (software- SkanIt Software 2.4.5 RE for Varioskan Flash). The ROS is represented as percentage of control (100%) (Hašková et al., 2011; Park et al., 2014; Ozbek et al., 2015).

### 2.8 *In- vivo* assessment of cardioprotective activity

All of the studies were carried out by following international standards for the use and care of experimental animals. Thirty Wistar albino rats (male), 10–12 weeks old, were bought from M/S Chakraborty Enterprise in Kolkata. The Institutional Animal Ethics Committee granted the study approval (IAEC no. IASST/2018/07). All of the animals were kept in tidy, empty plastic cages. The room was kept at a constant temperature of 24 ± 3 °C, with a relative humidity of 45 ± 5% and a cycle of light and dark, lasting 12 h. They were fed on standard rat chow and had unrestricted access to food and water *ad libitum*.

#### 2.8.1 Acute toxicity study

Six Wistar albino rats (both male and female) were chosen at random for the acute toxicity research, and their weights were noted. Following a single dosage of the GC extract at a dose of 2000 mg/kg body weight (BW) after an overnight fast in which they had only access to water, the animals were monitored continuously for the following 4 hours and kept in polypropylene cages under the same circumstances as the experimental animals, who had unlimited access to food and water. The animals were monitored for any behavioural abnormalities, toxicity, or mortality for the following 14 days in accordance with OECD (Organisation for Economic Co-operation and Development) 423 recommendations. The dose is deemed harmful if death is 2/3 or greater; if mortality is 1/3 or less, the experiment was to be repeated. If the mortality continued, lowering the dose was to be considered (Sarma et al., 2016).

#### 2.8.2 Development of ISO-induced MI experimental model

For the development of MI in experimental animals, isoproterenol is dissolved in normal saline and a dose of 85 mg/kg BW is injected into the animals subcutaneously on the 28th and 29th days of the experiment, at an interval of 24 h (Rajadurai and Prince, 2006; Ojha et al., 2012; Hassan et al., 2017).

#### 2.8.3 Experimental layout

After 7 days of acclimatization, the thirty rats (160–180 g) were distributed randomly into five groups with six animals each. They were pretreated with GC extract (prepared into a suspension in 0.3% carboxy methyl cellulose) at a dose of 100 and 200 mg/kg BW, every alternate day for 30 days. The experimental layout is as under-

A. Group I/IG1 - Normal control- Without ISO injection or GC.
B. Group II/IG2 - Disease control or ISO injected on the 28th and 29th days.
C. Group III/IG3 - Treated with standard drug (Atenolol) ISO + Atenolol - 5 mg/kg BW, every alternate day, for 30 days in 0.3% CMC.
D. Group IV/IG4 - Treated with 100 mg/kg BW GC- ISO + GC100.
E. Group V/IG5 - Treated with 200 mg/kg BW GC- ISO + GC200.

The ECG of all the animals were recorded after 2^nd^ injection of ISO and after recording ECG all the animals were euthanized on the same day after 24 h of 2^nd^ ISO injection with a combination of ketamine (87 mg/kg BW) and xylazine (13 mg/kg BW) injected intraperitoneally (Van Pelt, 1977) after fasting with just water for 12 h. Blood was collected for both serum and plasma, while heart tissues were collected after being washed with ice-cold physiological saline and stored at -80 °C for further biochemical and histopathological evaluation. Also, at the time of sacrifice, heart weight, body weight, and tail lengths of respective animals were recorded (Anosike & Cajetan, 2015; Shaikh et al., 2019).

#### 2.8.4 Cardiac hypertrophy parameters

The heart weight (mg), heart weight index [heart weight/body weight, (mg/g)] and heart tail index [heart weight/tail length (mg/cm)] were considered the preliminary indicators for hypertrophy in cardiac tissues. After thoroughly cleaning the heart in ice-cold saline and drying it on blotting paper, the heart’s weight was recorded at the time of dissection (Syed et al, 2016, Saleem et al., 2018).

#### 2.8.5 Analysis of electrocardiogram

At the time of recording ECG, the rats were anaesthetized using ketamine hydrochloride (100 mg/kg BW) *via* intraperitoneal injection (Kannan & Quine, 2011). Using three 12 mm long needle 29 gauge electrodes, Animal Bio Amp and PowerLab 8/35 (AD Instruments, Australia) ECG was done and the results were analysed using LabChart Pro software (N = 4). The changes in heart rate (bpm), RR- interval, P- duration, QRS interval, QT interval (ms), and ST height (mV) were recorded.

#### 2.8.6 Cardiac injury marker estimation

The following enzymes, viz. CK- MB (Creatinine kinase–MB isozyme), CK- NAC (Creatinine kinase-N-acetyl-L-cysteine), LDH (Lactate dehydrogenase), SGOT (serum glutamic oxaloacetic transaminase/AST), SGPT (serum glutamic pyruvic transaminase/ALT) and ALP (alkaline phosphatase) which are important diagnostic markers of cardiac injury were estimated from the serum using commercially available kits from Coral Clinical Systems (Tulip Diagnostics) (Panda et al., 2017). CK-MB levels were also measured from cardiac tissue extracts with the help of an ELISA kit (CAT IT7209, G- Biosciences) and cardiac troponin-I from serum (ELISA, CAT IT6638, G-Biosciences).

#### 2.8.7 Estimation of a lipid profile

The estimation of total cholesterol, triglyceride, LDL-C, and HDL-C have been estimated using a biochemistry analyzer (Selectra Junior, Vital Scientific) while VLDL- C was calculated using Friedwald’s formula-

$$VLDL-C=\frac{Triglycerides}{5}$$

(Friedwald et al., 1972; Panda et al., 2017). Further the HMG CoA (β-Hydroxyl ß-methylglutaryl- CoA) reductase activity was determined from the liver tissue homogenate by determining the HMG CoA/Mevalonate ratio following the protocol of Rao & Ramakrishnan, 1975.

#### 2.8.8 Myocardial oxidative stress parameters

For these assays, 10% (w/v) heart tissue extract in 0.05 M ice-cold phosphate buffer (pH 7.4). The estimation of lipid peroxidation levels was done using the TBARS estimation method described by Ohkawa et al., 1979 using the tissue extract directly without centrifugation. For SOD, GSH and catalase estimation, the tissue extract was centrifuged at 15,000 rpm for half an hour at 4 °C and was done using the methods described by Marklund and Marklund, 1974, Goth, 1991 respectively.

#### 2.8.9 Serum NO and myocardial iNOS/NOS2 levels

The serum NO was determined following the protocol as described by (Green et al., 1982). The iNOS/NOS2 levels were detected using an ELISA kit from G-Biosciences (CAT. IT6627).

#### 2.8.10 Inflammatory cytokines

The estimation of inflammatory cytokines TNF-α, IL- 1β, IL-6 and IL-10 were measured from serum and cardiac tissue extracts using ELISA kits following the manufacturer’s protocol (CAT: IT7716, IT7444, IT17833, IT18454 respectively, G- Biosciences).

#### 2.8.11 Histopathological analysis

After dissection, heart tissues were preserved in 10% neutral buffered formalin and were processed for histopathological analysis by embedding in paraffin. All tissues were sectioned with 5 µm thickness and stained with Haematoxylin- Eosin for general architecture and Masson’s Trichome for collagen deposition (blue colored) and observed for histoarchitectural changes. The images have been taken using a Zeiss Primo Star Phase contrast microscope installed with Zeiss Zen 3.3 blue edition software, scale 10 μm, magnification ×400.

### 2.9 Molecular docking

Human iNOS Reductase and Calmodulin Complex (PDB: 3HR4) and human TNF-alpha in complex with 2-[5-(3-chloro-4-{[(1R)-1-(2-fluorophenyl)ethyl]amino} quinolin-6-yl)pyrimidin-2-yl]propan-2-ol (PDB: 7JRA) were obtained from RCSB protein data bank and refined structural corrections carried out. The molecules were optimized and protein-ligand molecular docking was performed in extra precision mode. The molecular docking studies were carried out by following the standard procedures recommended by Schrodinger (Elancheran et al., 2019; Maruthanila et al., 2022).

### 2.10 Statistical analysis

The statistical analysis was done using Graphpad Prism Version five software. All data are expressed as mean ± standard deviation. The statistical significance is calculated *via* one-way ANOVA, with Tukey’s post hoc test for the biochemical data, and Dunnet’s multiple comparison tests for cell culture and animal experiment data. The results have been expressed in *p* < 0.05, 0.01, and 0.001 for statistical significance.

## 3 Results

### 3.1 Phytochemical investigation

After fractionating the crude extract (GPC), the obtained percentage yield for hexane and chloroform fractions was 1% and 8.2%, respectively. The ethyl acetate fraction amounted to about 64%, while the methanol fraction was about 20%. The total phenolic content assay revealed that GC (257.833 ± 7.507 mg GAE/g) had the highest phenolic content, followed by GH, GPC, GE, and GM in descending order among the crude and fractions (Supplementary Table S1). In comparison with crude extract, the phenolic content of GC and GH were significantly higher, followed by GE and GM. In between GC and GH, GC have substantially higher phenolic content (significantly higher at *p* < 0.05).

### 3.2 UHPLC-ESI orbitrap MS/MS analysis

**FIGURE 1:**
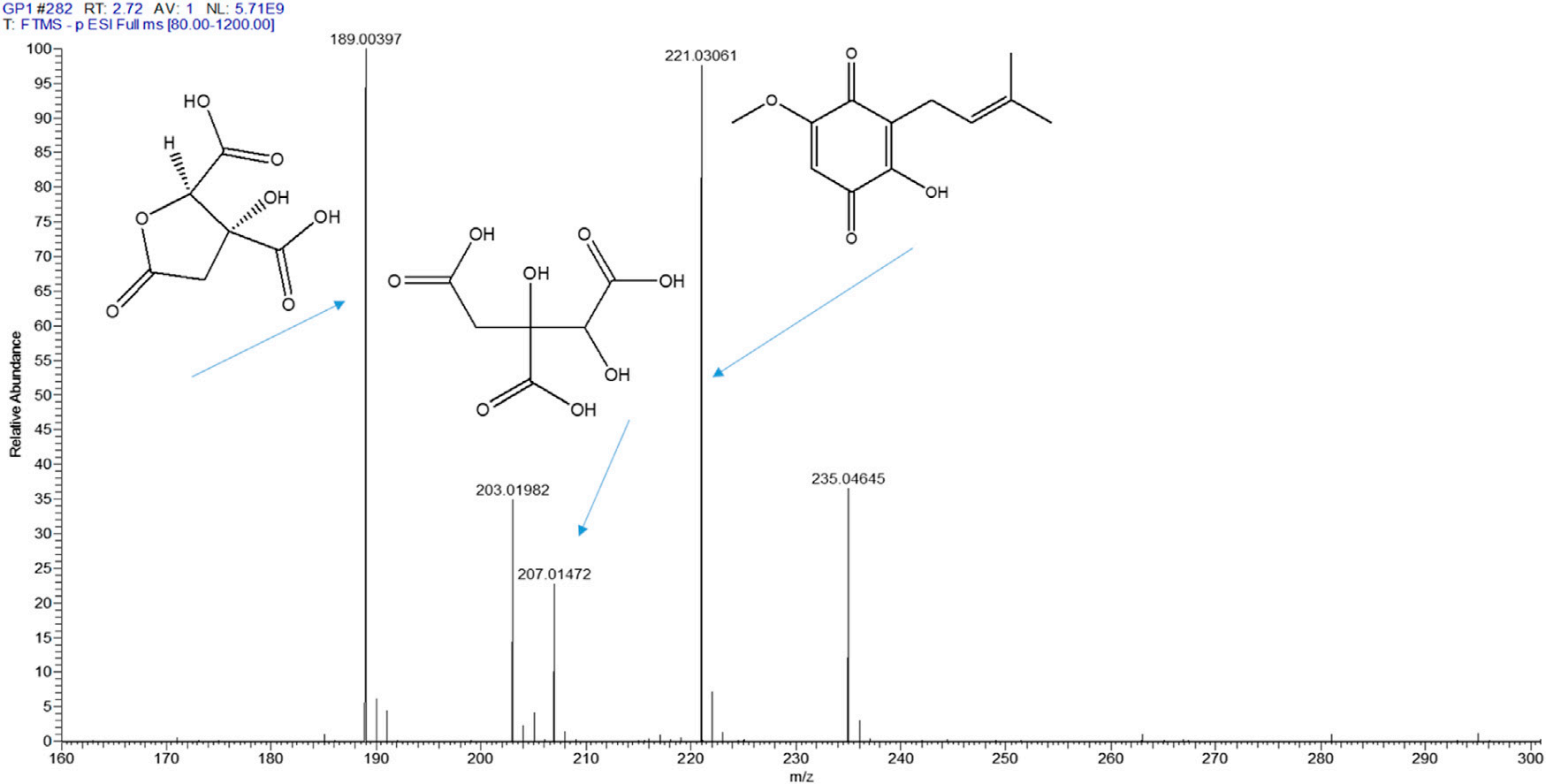
Identification of compounds from GP extract.

UHPLC-ESI-MS/MS was used for the screening and identification of crude extract (GPC) and the fractions. It shows that intense peaks were observed between 0 and 10 min. By comparing the GP extract mass, retention time, and λ max to those of the reference compounds and preceding literature publications, peak identification was carried out. hydroxycitric acid, hydroxycitric acid lactone, and parvifoliquinone were identified from GP extract with the respective mass [M-H]^+^, i.e. 189.00397, 207.01472 and 221.03061 respectively (Figure 1). Also, Hydroxycitric acid, GB-1a, Garcinone A, 9-Hydroxycalabaxanthone, Chlorogenic acid, and Garcinol were previously reported in our study.

### 3.3. *In- vitro* screening of antioxidant potential

Polyphenols are the most abundant secondary metabolites derived from plants which have a common structural skeleton with one or more aromatic rings with single or multiple hydroxyl substitutions. These compounds, have immense antioxidant and therapeutic potential (Swallah et al., 2020). Multiple studies reported the antioxidant properties of various plant extracts rich in polyphenolic compounds in amelioration of diseases like diabetes, cardiovascular, Alzheimer’s, and intestinal diseases (Stagos, 2019). The following assays take into account the free radical scavenging activity and metal-reducing power assays as a measure to assess the *in vitro* antioxidant potential of the extracts and fractions of this study.

#### 3.3.1 DPPH and ABTS radical scavenging assay

The results obtained from these two assays demonstrated the radical quenching capacity of the extracts, and GC showed significantly higher scavenging activity when compared to GPC and other extracts. The IC_50_ for the DPPH assay with GC is 31.81 ± 1.4253 μg/ml, which is also significantly lower (*p* < 0.05), exhibiting enhanced radical scavenging activity when compared to GH (62.98 ± 1.8344 μg/ml), followed by GPC, GE, and GM. When compared to GPC (318.66 ± 2.1195 μg/ml), GC and GH had significantly lower IC_50_, thereby having better activity in comparison. In contrast, GE and GM with significantly higher IC_50_ showed much less scavenging activity. In the ABTS assay, a similar pattern was established with GC exhibiting excellent radical scavenging activity with the lowest IC_50_ (36.31 ± 4.7099 μg/ml), which was significantly lower (*p* < 0.05) than that of GH (51.53 ± 2.4623 μg/ml) and the other extracts as well. Similarly, when compared to GPC (373.78 ± 8.3045 μg/ml), GC and GH showed better radical scavenging activity, whereas GM and GE had significantly inferior activity in comparison. The results are in detail in Supplementary Table S2.

#### 3.3.2 FRAP and PMD assay

Both of these assays comprise a similar chemical reaction involving the reduction of metal ions [Fe^3+^ to Fe^2+^ and Mo (VI) to Mo (V)], also taken as a parameter of antioxidant activity. In FRAP as well as in PMD assay, the EC_50_ of GC (FRAP: 216.14 ± 13.8743 μg/ml; PMD: 269.61 ± 10.9951 μg/ml) was significantly lower when compared to all crude as well as other fractions, which is an indication of its excellent reducing power and in turn the fraction’s antioxidant potential. Next, in comparison GPC, GE, and GM, GH exhibited better activity with significantly lower EC_50_ (FRAP: 414.24 ± 5.9386 μg/ml; PMD: 385.31 ± 3.9779 μg/ml). In between GC and GH, GC was more active than GH (Supplementary Table S3).

### 3.3.3 *In- vitro* lipid peroxidation assay

In this assay, the iron-ascorbate system induces lipid peroxidation *in vitro* conditions *via* hydroxyl radical generation. The extent of inhibition of this reaction is considered a measure of the antioxidative potential. Upon screening all the extracts, GC exhibited better lipid peroxidation inhibitory activity with a significantly lower IC_50_ (310.76 ± 11.6898 μg/ml), as compared to GH (614.92 ± 7.3873 μg/ml). Also, the IC_50_ of GPC, GE, and GM was significantly higher than that of GC, as mentioned in Supplementary Table S4. Following the same pattern as the assays mentioned earlier, GC also exhibits good antioxidant capacity as it actively inhibits the *in vitro* lipid peroxidation in the heart tissue samples.

### 3.4 *In vitro* assessment of cardioprotective activity in cardiomyocytes

#### 3.4.1 Microscopic observation

After careful microscopic observations, the ISO-treated cells lost their standard elongated shape, and most of them were rounded off and lost their adherent properties. In contrast, the GC-treated cells retained their normal morphology and attachment properties, resembling the untreated control cells (Supplementary Figure S1).

#### 3.4.2 Cell viability assay

On treating H9c2 with just different concentrations of ISO, there was a significant gradual dose-dependent decrease in cell viability from 10 to 100 µM concentration compared to the untreated control. With about 30% cell death, 25 µM dose of ISO was used for the rest of the cell-based assays. After treatment with just GC in H9c2 cells for 24 h, no significant (*p* < 0.001) changes were found up to 100 μg/ml compared to normal control cells. There was a decline in cell viability at a concentration of 200 μg/ml, which was significantly lower (*p* < 0.001) than the control. Therefore, safe doses lower than 10 μg/ml are selected for further studies (Supplementary Figure S2).

**FIGURE 2:**
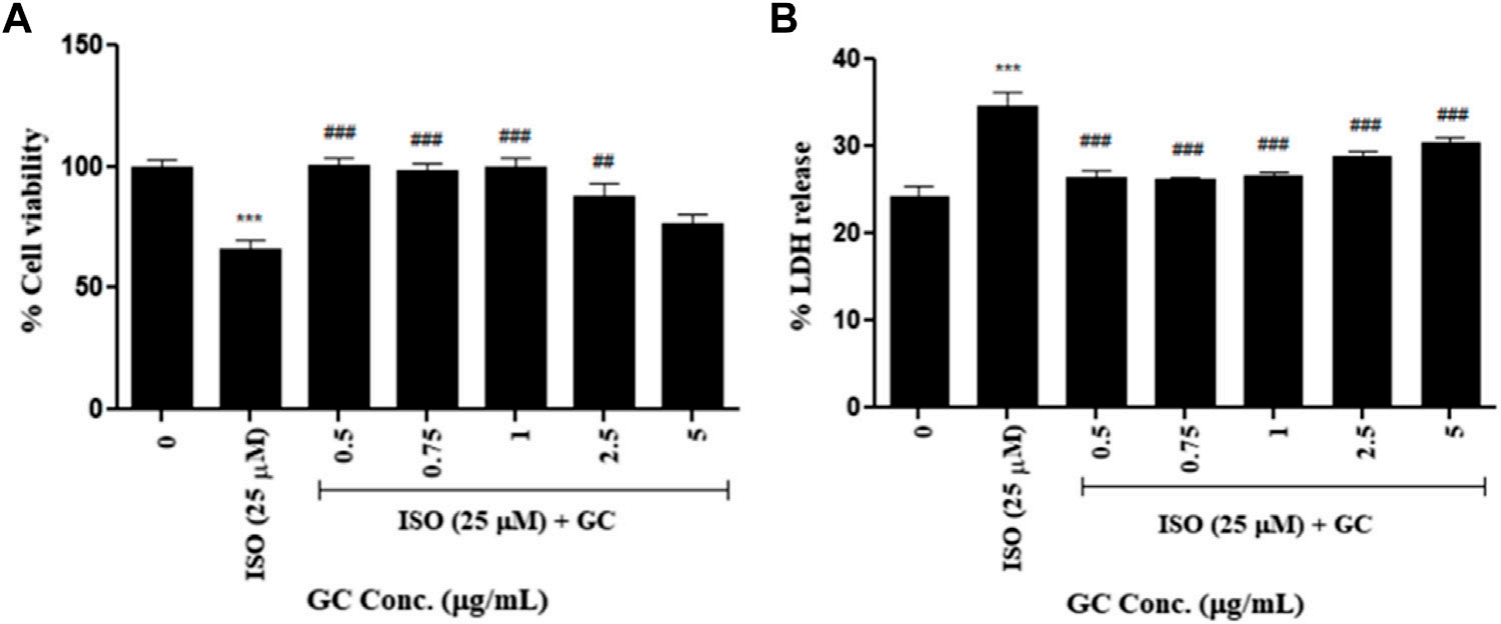
Cell viability assay- **(A)** MTT and **(B)** LDH release assay in ISO (25 µM) induced cardiotoxicity model in H9c2 cell line with GC pretreatment at different concentrations (µg/ml). Data expressed as mean ± SD for groups of three observations. Statistically significant *** *p* < 0.001 compared to control group and ### *p* < 0.001 compared to ISO group as calculated by one-way ANOVA followed by Dunnet’s multiple comparison tests).

In the ISO-induced cardiotoxicity model, for just ISO treated group cell viability decreased significantly (*p* < 0.001) when compared to untreated control and it was seen that pretreatment with GC at concentrations 0.5, 0.75, 1, 2.5 μg/ml significantly (*p* < 0.001) increased the cell viability against ISO treatment which were not significantly different when compared to control cells, thereby indicating the cardioprotective effect of GC against the cardiotoxic effects of ISO (Figure 2). Although, interestingly the lower doses were found to have better protective activity than the higher doses. The data is represented in Figure 2A.

#### 3.4.3 LDH release assay

This assay showed that LDH release was significantly higher (*p* < 0.001) in the ISO treated group than in the untreated control. A significant reduction in the same (*p* < 0.001) was recorded with GC pretreatment at multiple concentrations of 0.5–5 μg/ml when compared to ISO treated group. The levels of LDH with 0.5, 0.75, and 1 μg/ml of GC treatment were not significantly different from that of the normal cells (*p* < 0.001) (Figure 2B).

#### 3.4.4 Intracellular ROS detection

**FIGURE 3:**
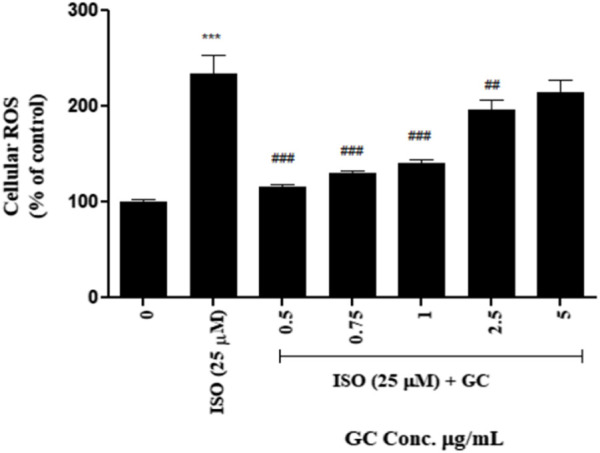
Intracellular ROS detection assay in ISO (25 µM) induced cardiotoxicity model in H9c2 cell line with GC pretreatment at different concentrations (µg/ml). Data expressed as mean ± SD for groups of three observations. Statistically significant *** *p* < 0.001 compared to control group and ### *p* < 0.001 compared to ISO group as calculated by one-way ANOVA followed by Dunnet’s multiple comparison tests).

After measuring the fluorescence intensity from H_2_DCF-DA in the treated cells vs. untreated control, it was observed that ROS was significantly increased (*p* < 0.001) in ISO-treated cells after 12 h of treatment. With GC pretreatment at 0.5, 0.75, 1, and 2.5 μg/ml, it was seen that there was a steep significant reduction in ROS (*p* < 0.001) when compared to the ISO group. The 5 μg/mL GC treatment showed some reduction in ROS but was not significantly different from ISO group. Also, among the GC-treated groups, 0.5, 0.75, and 1 μg/ml showed a better reduction in ROS than in 2.5 and 5 μg/ml (Figure 3).

### 3.5 *In- vivo* assessment of cardioprotective activity

#### 3.5.1 Acute toxicity studies

In the first 4 hours after administration of the drug (GC, 2000 mg/kg BW) orally, none of the animals died or demonstrated any abnormal behaviour on their part. There was no mortality even in the following 2 weeks interval where all the rats survived without any evident clinically abnormal fluctuations in their behaviour. With this, the drug safety was confirmed for 2000 mg/kg BW. Two subsequent doses, 2/10th and 1/10th of this dose, were decided on for further studies.

#### 3.5.2 Cardiac hypertrophy parameters

**FIGURE 4:**
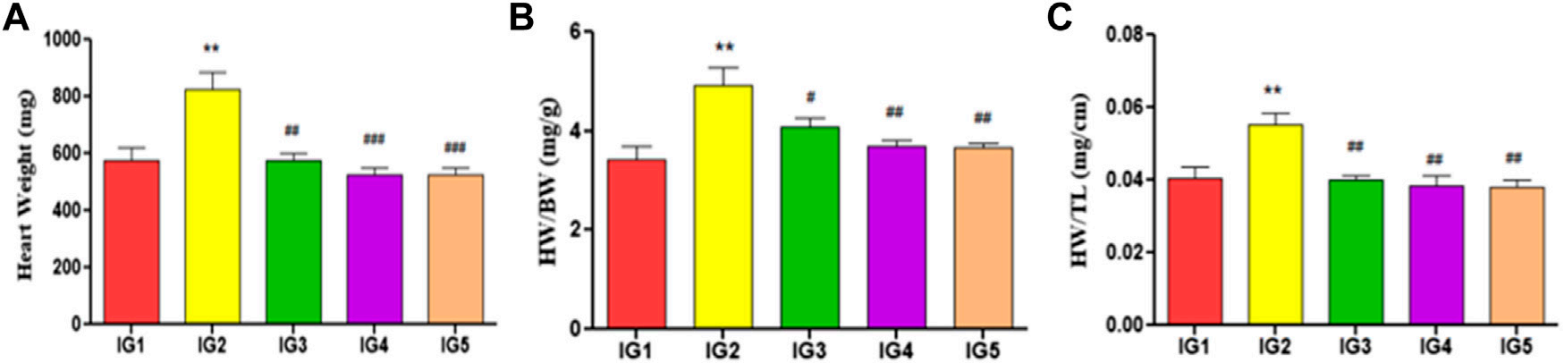
Effect of ISO and GC pre-treatment on cardiac hypertrophy parameters-**(A)** Heart weight (mg), **(B)** Heart weight index (mg/g) and **(C)** Heart tail index (g/cm). Results are expressed as mean ± SD for groups of three observations. Statistically significant at *** *p* < 0.001, ** *p* < 0.01, * *p* < 0.05 as compared to IG1 and ^###^ *p* < 0.001, ^##^ *p* < 0.01, ^#^ *p* < 0.05 when compared to IG2 as calculated by one-way ANOVA followed by Dunnet’s multiple comparison tests.

The body weight gain did not vary significantly between the groups over the experimental period (Supplementary Table S5). However, a significant rise in heart weight (*p* < 0.01) in IG2 was observed as compared to the normal control group, while in Atenolol treated group, a significant decrease (*p* < 0.01) was noted when compared to IG2. There was a significant decrease in both the GC pretreatment groups (*p* < 0.001) compared to the ISO group in terms of heart weight. As a measure of hypertrophy of the heart, the heart weight index (mg/g) and heart tail index (mg/cm) were significantly increased (*p* < 0.01) in IG2 when compared to IG1. With the GC pretreated group, a significant decrease in both ratios was recorded at *p* < 0.01 compared to IG2, with no significant difference when compared to IG1 (Figure 4).

#### 3.5.3 Analysis of parameters of electrocardiogram

**FIGURE 5:**
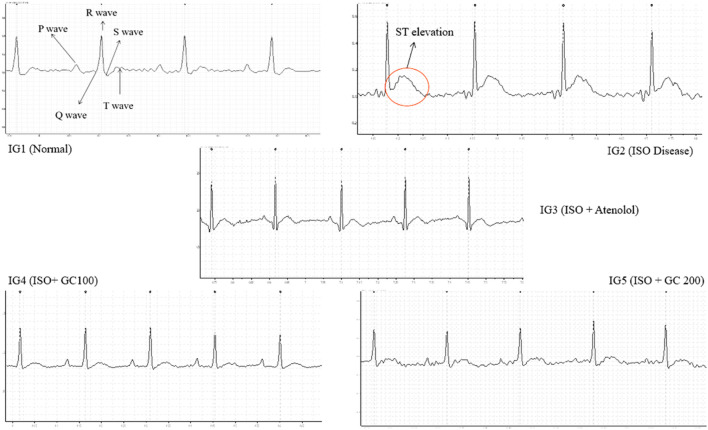
Representative electrocardiograph recordings of all the experimental groups, with evident ST elevation found in ISO treated disease group.

From the data obtained (Table 1), a sharp and significant increase in heart rate (*p* < 0.01), ST height or elevation (*p* < 0.001), QRS and QT interval (*p* < 0.01) in the ISO treated disease group was observed along with a significant decrease in R-R interval (ms) and P- duration (ms) on comparison with the normal control group (Figure 5). The ST elevation is often treated as a marker of myocardial infarction pathologically, while the other parameters are an indication of myocardial injury caused due to ISO injection (Jain et al., 2018). Upon treatment with Atenolol, there was a significant (*p* < 0.01) decrease in the heart rate and P duration (0.001). There was also a visible increase in the R-R interval, although not statistically significant.

**TABLE 1:** Electrocardiographic findings of experimental groups depicting the effect of ISO and GC pretreatment. Results are expressed as mean ± SD for groups of three observations, Statistically significant at *** *p* < 0.001, ** *p* < 0.01, * *p* < 0.05 as compared to IG1 and ^###^ *p* < 0.001, ^##^ *p* < 0.01, ^#^ *p* < 0.05 when compared to IG2 as calculated by one-way ANOVA followed by Dunnet’s multiple comparison tests.

Groups	HR (bpm)	R-R interval (ms)	P duration (ms)	QRS interval (ms)	QT interval (ms)	ST height (mV)
IG1	415.8 ± 3.84	144.37 ± 1.37	25.60 ± 2.84	15.76 ± 1.41	51.91 ± 4.07	0.0262 ± 0.005
IG2	535.7 ± 29.47 **	114.56 ± 2.87 *	10.66 ± 1.26 *	39.18 ± 8.28 **	90.54 ± 10.71 **	0.1785 ± 0.015 ***
IG3	434.4 ± 14.16 ^##^	138.5 ± 4.92	27.63 ± 1.26 ^###^	15.91 ± 2.72 ^###^	77.92 ± 2.87	0.0371 ± 0.0106 ^###^
IG4	346.07 ± 27.76 ^###^	174.5 ± 14.01 *^,###^	23.89 ± 3.34 ^##^	13.48 ± 1.50 ^###^	61.27 ± 3.22 ^##^	0.0254 ± 0.007 ^###^
IG5	356.73 ± 30.05 ^###^	169.5 ± 14.73 ^###^	22.81 ± 2.61 ^##^	13.56 ± 2.84 ^###^	64.86 ± 4.44 ^##^	0.0274 ± 0.007 ^###^

Also, in IG3, there was a significant decrease (*p* < 0.001) in QRS interval and ST elevation compared to IG2. The interesting finding from this assessment is that GC pretreatment at both concentrations could protect the heart against ISO-induced damage. It was evident from the significantly lowered heart rate and ST elevation (*p* < 0.001), QRS (*p* < 0.001) and QT interval (*p* < 0.01), and also from the significant increase in the R-R interval (*p* < 0.001), P- duration (0.01) when compared to IG2. These results were comparable to the ECG recording of the normal control group (Table 6). Hence, it will be suitable to infer that GC pretreatment significantly reverses the adverse effects of ISO.

#### 3.5.4 Cardiac injury marker estimation

**FIGURE 6:**
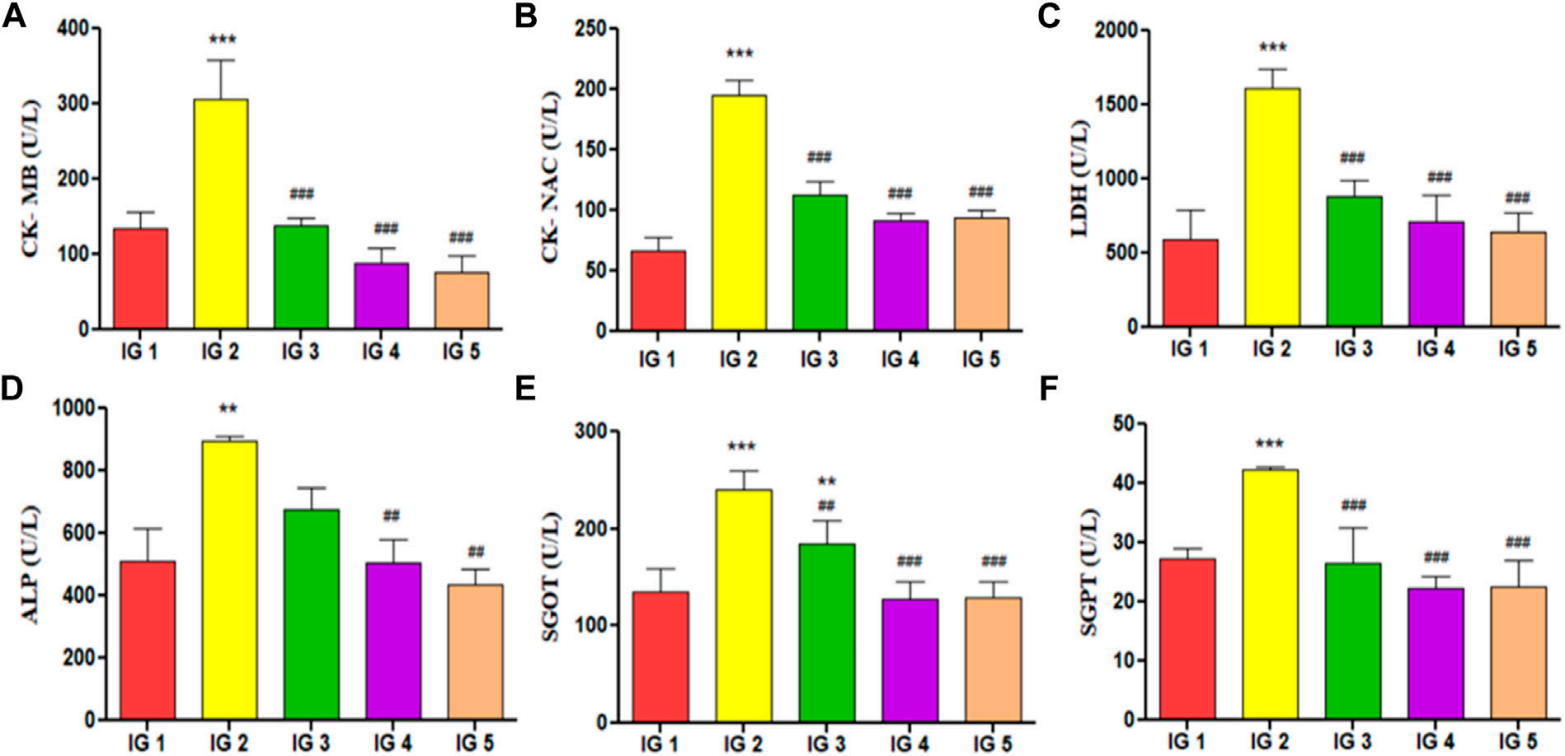
The effect of ISO and GC pretreatment on cardiac injury biomarker enzymes in serum of the experimental groups. Results are expressed as mean ± SD for groups of three observations, statistically significant at *** *p* < 0.001, ** *p* < 0.01, * *p* < 0.05 as compared to IG1 and ^###^ *p* < 0.001, ^##^ *p* < 0.01, ^#^ *p* < 0.05 when compared to IG2 as calculated by one-way ANOVA followed by Dunnet’s multiple comparison tests. **(A)** CK-MB, **(B)** CK-NAC, **(C)** LDH, **(D)** ALP, **(E)** SGOT, **(F)** SGPT.

In this study, the following cardiac injury marker enzymes with diagnostic importance have been estimated from serum, *viz.* CK- MB, CK- NAC, LDH, SGOT, SGPT, and ALP (Figure 6). In the results obtained, it was evident that all the cardiotoxicity marker enzymes were significantly elevated (*p* < 0.001 and *p* < 0.01) with just ISO treated disease group when compared to normal control. With atenolol treatment, these parameters were found to be significantly (*p* < 0.001) reduced to near-normal levels in all except ALP. GC pretreatment could also substantially lower all these marker enzymes (*p* < 0.001 and *p* < 0.001) in both concentrations compared to IG2. These levels were much in the range of the normal control group (Figure 8). In addition to these, CK- MB levels were estimated from heart tissue homogenates *via* ELISA to understand its tissue-specific expression levels in all the experimental groups. The results showed a 3-fold significant elevation in the level (*p* < 0.01) of CK- MB in IG2 compared to IG1. In contrast, with atenolol treatment, the expression was significantly suppressed (*p* < 0.01) compared to IG2. A similar trend was observed in GC pretreated groups, where the CK- MB expression levels were significantly reduced (*p* < 0.01) and in the range of the normal untreated control group when compared to IG2 (Table 2). These results strongly indicate that GC pretreatment successfully protected the myocardial tissues against ISO-induced cardiotoxic injury.

**TABLE 2:** Effect of ISO and GC pre- treatment on CK- MB (ng/ml) levels in the heart tissue homogenates and cardiac troponin I (cTnI) in serum.

Groups	IG1	IG2	IG3	IG4	IG5
CK- MB (ng/mg protein)	0.27 ± 0.1381	1.09 ± 0.3099**	0.29 ± 0.1843^##^	0.32 ± 0.1878^##^	0.21 ± 0.1469^##^
Cardiac Troponin I (cTnI, pg/mL)	51.78 ± 2.9788	107.08 ± 9.0561 ***	79.34 ± 5.8476 ^###^	60.51 ± 4.0515 ^###^	70.23 ± 3.4798 ^###^

(Results are expressed as mean ± SD for groups of three observations, statistically significant at *** *p* < 0.001, ** *p* < 0.01, * *p* < 0.05 as compared to IG1 and ### *p* < 0.001, ## *p* < 0.01, # *p* < 0.05 when compared to IG2 as calculated by one-way ANOVA followed by Dunnet’s multiple comparison tests.)

Moreover, the cardiac troponin I levels estimated from serum using ELISA revealed a significant (*p* < 0.001) 2.1-fold increase in the ISO treated disease group (IG2) compared to normal untreated control (IG1). With atenolol treatment, the situation improved significantly (*p* < 0.001) bringing the troponin I levels to near normal in IG3. With GC pretreatment, it was observed that IG4 with 100 mg/kg BW showed a better and significant (*p* < 0.001) decrease compared to IG5, which also recorded significant reduction in serum troponin I compared to the disease group. In both the groups, GC pretreatment significantly prohibited the ISO-induced increase in cardiac troponin I levels, thereby protecting the heart successfully.

#### 3.5.5 Estimation of lipid profile

The lipid profile of the ISO-treated group had significantly elevated levels of LDL-C (*p* < 0.05), triglycerides (*p* < 0.001), cholesterol (*p* < 0.01) as well as VLDL-C (*p* < 0.001) when compared to normal control. Also, IG 2 group had significantly depleted (*p* < 0.05) levels of HDL-C as well. In the Atenolol treated group, HDL-C level was only marginally increased, LDL-C and cholesterol were marginally decreased but were not statistically significant. In contrast, the triglycerides and VLDL-C levels were significantly reduced compared to IG2. With GC pretreatment, a significant increase in HDL-C was seen in both IG4 (*p* < 0.01) and IG5 (*p* < 0.05) when compared to IG2. In the triglycerides, cholesterol and VLDL-C levels, a sharp and significant decline (*p* < 0.001) was seen in both GC pretreated groups again compared to IG2 (Table 3). A significant decrease was also noted in LDL-C (IG4- *p* < 0.05 and IG5- *p* < 0.001) levels upon comparison with ISO treated group. The estimation of HMG CoA reductase activity *via* indirect method revealed that the ISO induced disease group, IG2 showed a significant (*p* < 0.01) 1.2-fold decrease in the HMG CoA/Mavalonate ratio, an indication of increased cholesterol synthesis compared to the normal IG1 group. In the GC pretreated groups, a significant (*p* < 0.01) increase in the HMG CoA/Mavalonate ratio was noted, indicating that GC could reduce overall activity of HMG CoA reductase activity.

**TABLE 3:** Effect of ISO and GC pretreatment on the lipid profile.

Groups	HDL-C (mg/dl)	LDL-C (mg/dl)	Triglycerides (mg/dl)	Cholesterol (mg/dl)	VLDL-C (mg/dl)	HMG CoA/Mavalonate
IG1	29.00 ± 6.633	14.75 ± 3.178	61.25 ± 6.449	38.70 ± 2.947	12.25 ± 1.290	1.32 ± 0.0744
IG2	18.50 ± 2.741*	21.98 ± 2.494 *	136.8 ± 13.82 ***	51.80 ± 2.947 **	27.35 ± 2.763 ***	1.12 ± 0.0306 **
IG3	20.75 ± 3.557 ^#^	20.70 ± 4.826 *	93.50 ± 4.359 ^###^	43.15 ± 1.196	18.70 ± 0.8718 ^###^	1.21 ± 0.1277
IG4	31.13 ± 3.983 ^##^	14.35 ± 1.816 ^#^	47.50 ± 7.594 ^###^	24.40 ± 3.667 ^###^	9.5 ± 1.519 ^###^	1.36 ± 0.0569 ^##^
IG5	29.85 ± 5.176 ^#^	13.03 ± 1.960 ^##^	51.00 ± 6.481 ^###^	28.28 ± 6.002 ^###^	10.20 ± 1.296 ^###^	1.33 ± 0.0849 ^##^

(Results are expressed as mean ± SD for groups of three observations, statistically significant at *** *P* < 0.001, ** *P* < 0.01, * *P* < 0.05 as compared to IG1 and ^###^ *P* < 0.001, ^##^ *P* < 0.01, ^#^ *P* < 0.05 when compared to IG2 as calculated by one-way ANOVA followed by Dunnet’s multiple comparison tests.)

#### 3.5.6 Myocardial oxidative stress parameters

**FIGURE 7:**
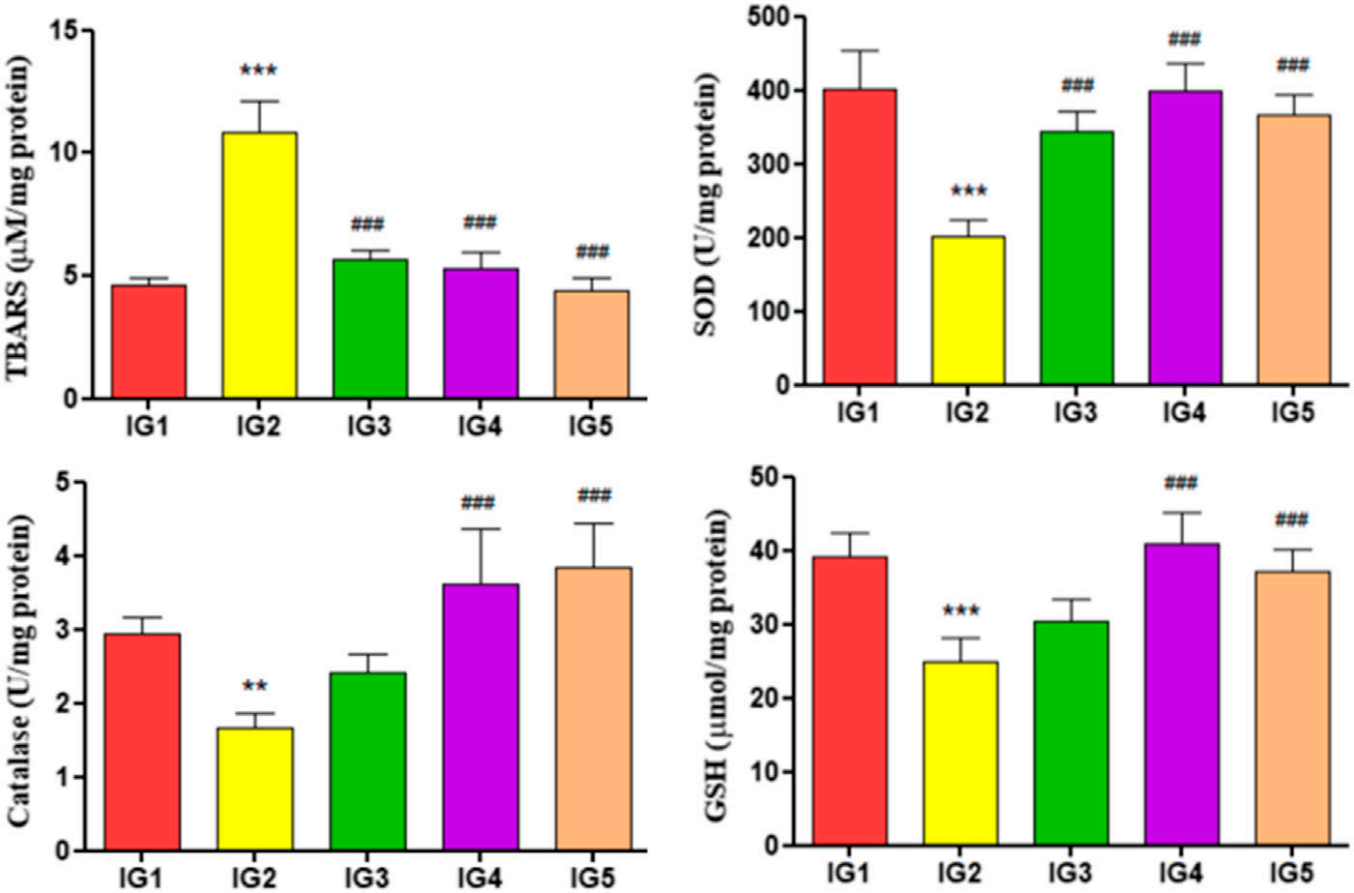
The effect of ISO and GC pretreatment on the myocardial endogenous antioxidants and lipid peroxidation levels in the experimental groups. Results are expressed as mean ± SD for groups of three observations, statistically significant at *** *p* < 0.001, ** *p* < 0.01, * *p* < 0.05 as compared to IG1 and ^###^ *p* < 0.001, ^##^ *p* < 0.01, ^#^ *p* < 0.05 when compared to IG2 as calculated by one-way ANOVA followed by Dunnet’s multiple comparison tests.

The endogenous antioxidants, *i.e.,* SOD, catalase, and GSH levels, were assessed in the heart tissue extracts for all the experimental groups and their lipid peroxidation status (Figure 7). The lipid peroxidation levels were detected by estimating TBARS in respective samples, and in the ISO-treated group, it was significantly elevated (*p* < 0.001) by 2.3-fold compared to IG1. In both the GC pretreated groups, however, the levels of TBARS were significantly reduced (*p* < 0.001) compared to IG2. It was also found that with ISO treatment in the IG2 group, the levels of SOD (*p* < 0.001), GSH (*p* < 0.001), and catalase (*p* < 0.01) were significantly reduced by 1.9-fold, 1.5-fold, and 1.7-fold respectively when compared to IG1. But with GC pretreatment, the levels of these endogenous antioxidants were returned to near-normal levels. They were found to be significantly reduced (SOD and GSH—*p* < 0.001 and catalase—*p* < 0.01 and *p* < 0.001) in both IG4 and IG5 when compared to ISO treated disease group.

#### 3.5.7 Serum NO and myocardial iNOS/NOS2

**FIGURE 8:**
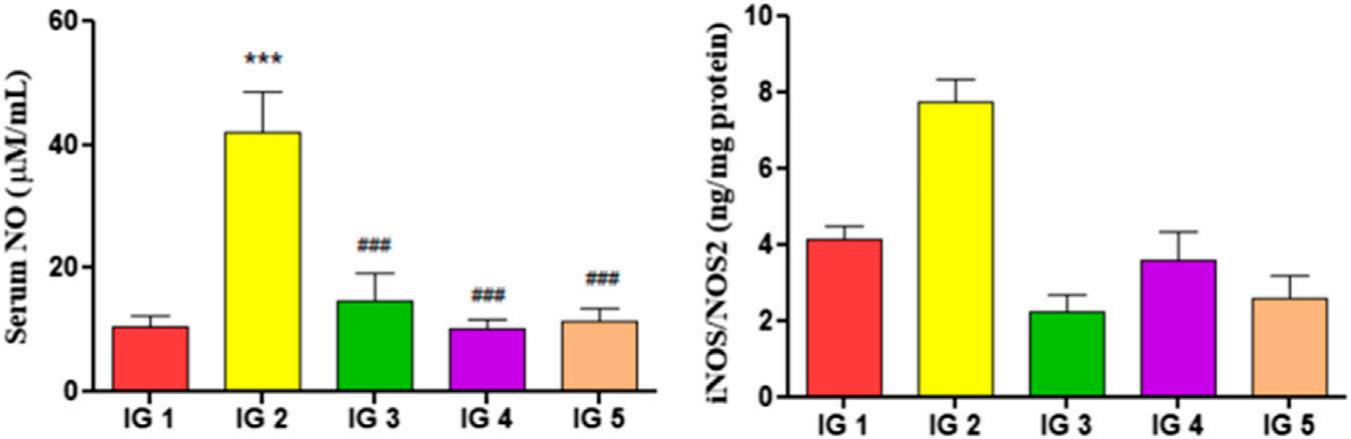
The effect of ISO and GC pretreatment on the serum NO and the iNOS/NOS2 levels in the myocardial tissue extracts of the experimental groups. Results are expressed as mean ± SD for groups of three observations, statistically significant at *** *p* < 0.001, ** *p* < 0.01, * *p* < 0.05 as compared to IG1 and ^###^ *p* < 0.001, ^##^ *p* < 0.01, ^#^ *p* < 0.05 when compared to IG2 as calculated by one-way ANOVA followed by Dunnet’s multiple comparison tests.

In ISO treated group IG2, the serum NO and iNOS/NOS2 (Figure 8) in the heart tissue extract were significantly elevated (*p* < 0.001 and *p* < 0.01) by 3.9-fold and 1.8-fold respectively, compared to the normal untreated control group. However, with GC pretreatment, these levels were significantly reduced (*p* < 0.001) in both groups compared to IG2.

#### 3.5.8 Inflammatory cytokines

**FIGURE 9:**
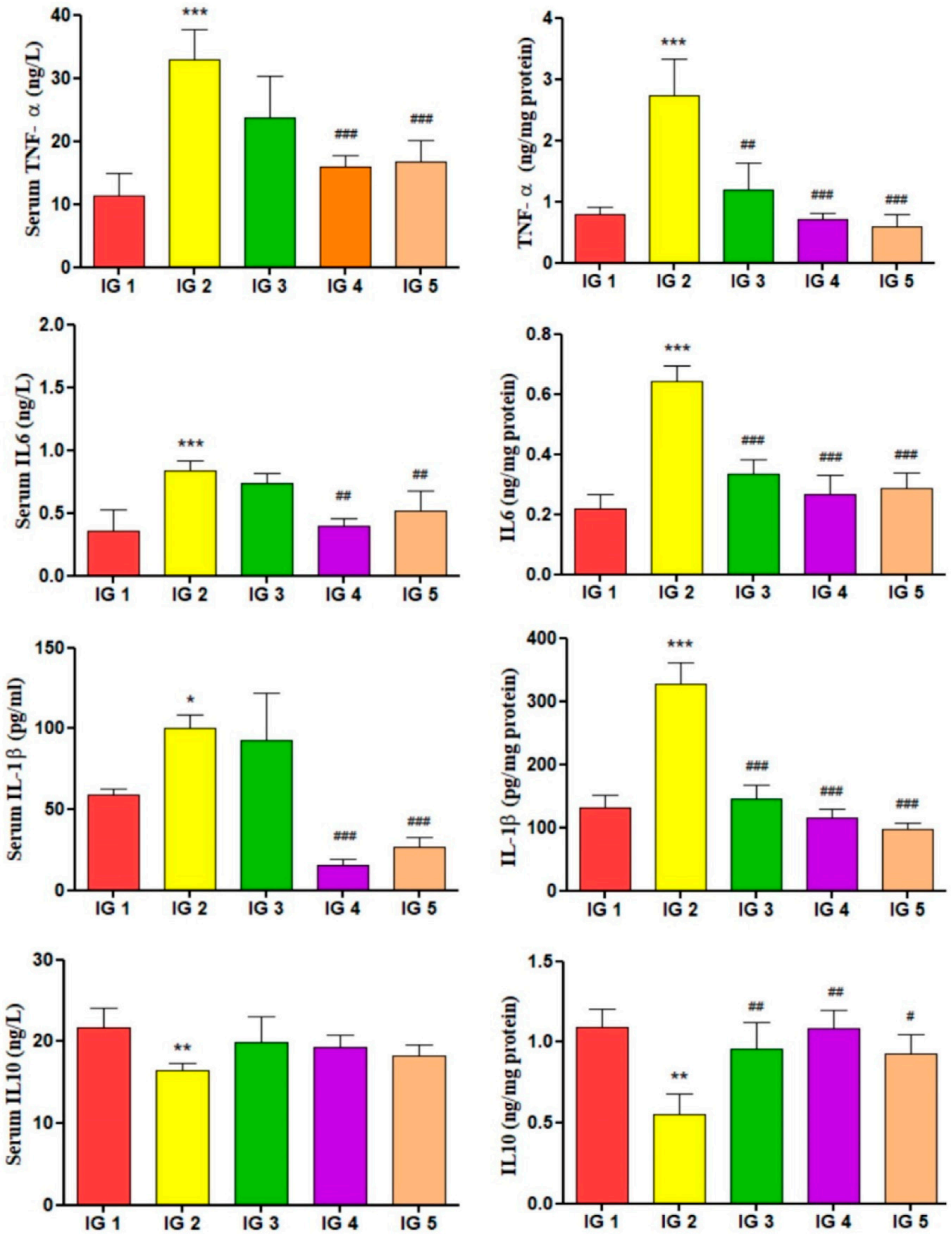
The effect of ISO and GC pretreatment on the inflammatory cytokines IL-6, TNF- α, IL- 1β, and IL- 10 levels in the myocardial tissue extracts of the experimental groups. Results are expressed as mean ± SD for groups of three observations, statistically significant at *** *p* < 0.001, ** *p* < 0.01, * *p* < 0.05 as compared to IG1 and ^###^ *p* < 0.001, ^##^ *p* < 0.01, ^#^ *p* < 0.05 when compared to IG2 as calculated by one-way ANOVA followed by Dunnet’s multiple comparison tests.

The inflammatory cytokines like IL-6, IL- 10, TNF- α, and IL- 1β were quantified using ELISA from myocardial tissue extracts and serum. When compared to the untreated control group, the levels of pro-inflammatory cytokines IL-6 (2.9-fold), TNF-α (3.4-fold), and IL- 1β (2.4-fold) were significantly elevated (*p* < 0.001), whereas the anti-inflammatory cytokine IL-10 was significantly (*p* < 0.01) reduced by 1.9-fold in the heart tissue extracts of the ISO treated group. In IG4, IL-6, TNF- α, IL- 6 (*p* < 0.001) was significantly reduced, and in IG5 as well, there was a significant decrease in all the pro-inflammatory cytokine levels (*p* < 0.01 and *p* < 0.001) compared to IG2. IL- 10 was significantly increased in both IG4 (*p* < 0.01) and IG5 (*p* < 0.05) when compared to IG2. A similar trend was observed for the inflammatory cytokines in the serum samples, but the results were more pronounced in the tissue samples. It was noted that the pro-inflammatory cytokines TNF-α, IL-6, and IL-1β, increased significantly (*p* < 0.001) in the IG2 group, while the anti-inflammatory IL-10 decreased significantly (*p* < 0.01) in IG2. With GC pretreatment (in IG4 and IG5) this situation was successfully corrected as all the cytokines were revived to the near normal state resembling IG1. It can be concluded from here that the anti-inflammatory action of GC aided in its cardioprotective effect. The detailed results are presented in Figure 9.

#### 3.5.9 Histopathological study

**FIGURE 10:**
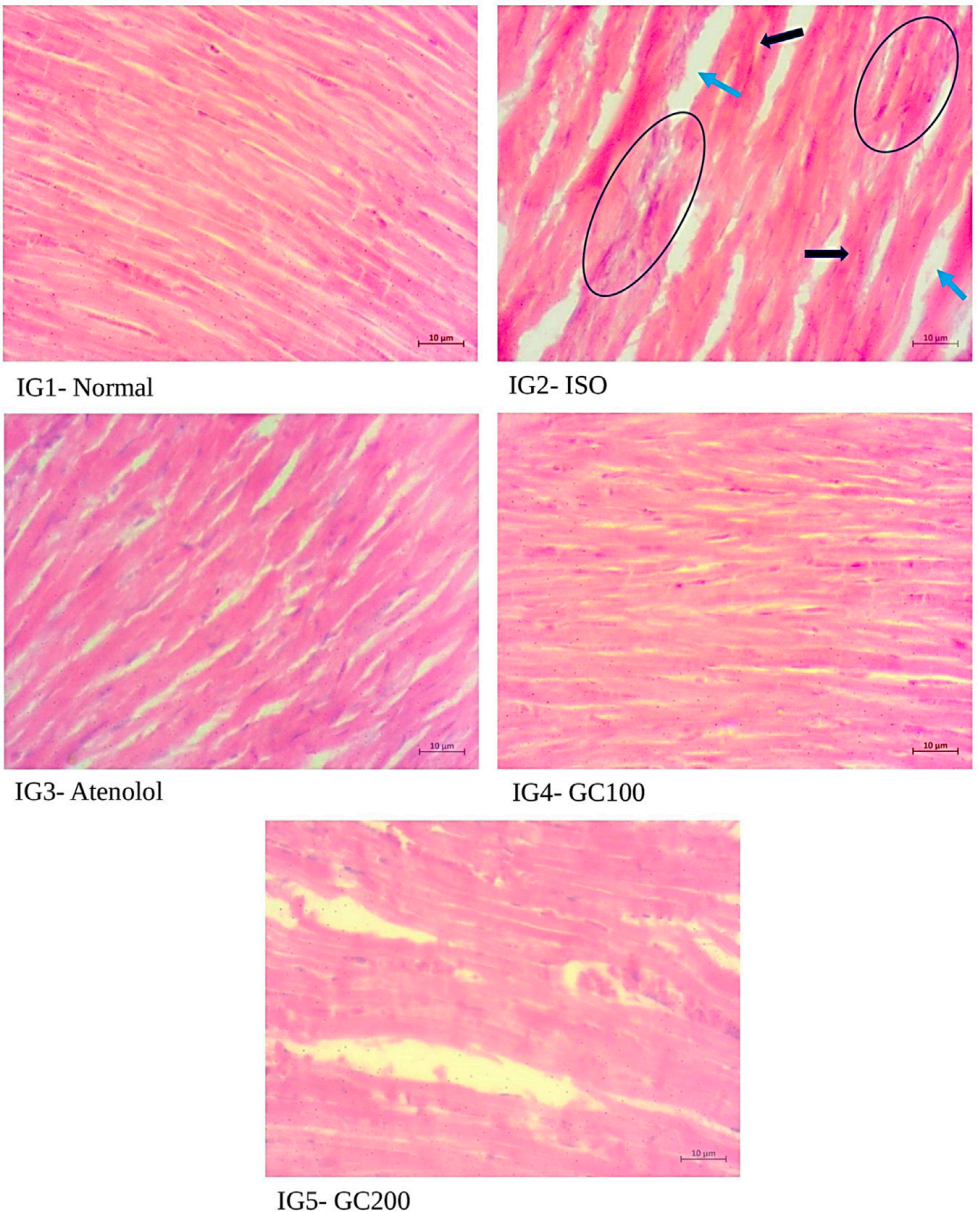
Histopathological analysis of H& E staining- a comparison with the normal, ISO treated and GC- pre- treatment experimental groups. Scale bar- 10 μm, Magnification ×400. (Blue arrow- infracts, black arrow- wavy myofibrils, circles—Necrotic region).

**FIGURE 11:**
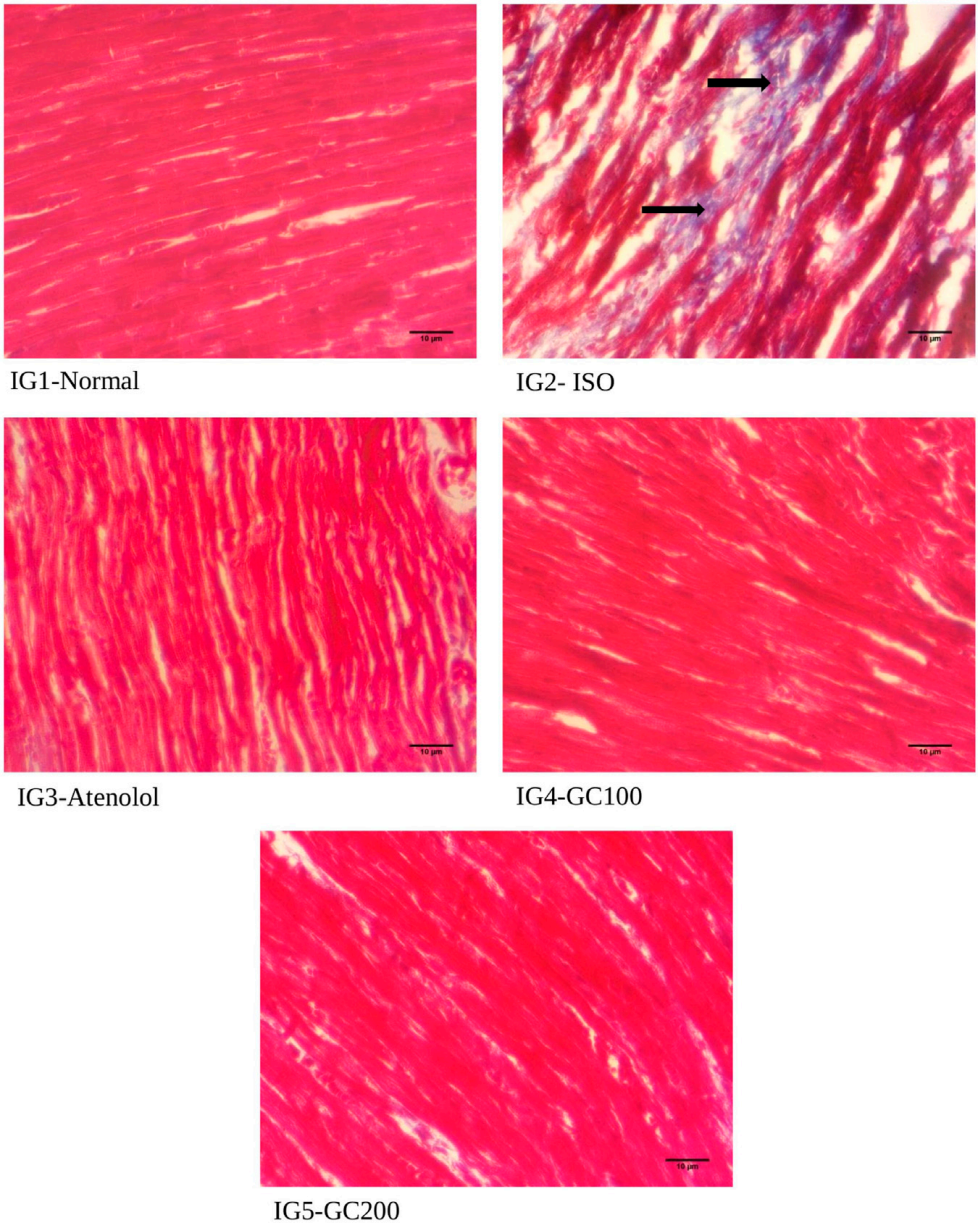
Histopathological analysis of Masson trichome staining- a comparison with the normal, ISO treated and GC- pre- treatment experimental groups. Scale bar- 10 μm, Magnification ×400. (Blue stained areas marked with black arrows show collagen deposition, an indication of interstitial fibrosis).

From the histopathological analysis with H& E staining (Figure 10) of heart tissues, it was evident that there was extensive damage to the muscle membrane, with an overall distorted myocardial architecture in the ISO treated group. It was also seen that there were visible gaps between the myofibrils, marked as ‘inf’ as they depict the infracts with the presence of wavy myofibrils (WMf) surrounding these infract regions, along with a few necrotic regions (N) in the disease group. All these changes marked the ISO induced extensive damage to the myocardial architecture. The normal heart tissues had well-aligned myofibrils arranged in a very compact packing without gaps and well-maintained myocardial membrane integrity without any distortions. The pretreatment with GC could restrict the histo- architectural damages to the heart tissues even after ISO treatment as was seen from the retained near normal packing of the myocardial fibers with intact membrane structure, the absence of necrotic lesions infracts in the tissue sections of IG4 and IG5. With Masson trichome staining (Figure 11), myocardial tissues appear red in colour while collagen stains blue, and were used to study the extent of collagen deposition (blue colored) in the heart tissues. It was seen that ISO treatment greatly increased the collagen deposition among the myocardial fibers, which is an indication of interstitial fibrosis, which was nearly non-existent in the normal untreated control group. These changes were greatly reduced with GC and atenolol treatment as a visible reduction in collagen deposition is seen in the respective groups. These results indicate that with the pre- treatment using GC fraction, the hearts of the rats were well protected against the damages inflicted after ISO injection. These results further emphasize the cardioprotective potential of GC.

### 3.6 Molecular docking

Molecular docking was performed on the compounds found from UHPLC-MS/MS and the binding interactions and binding energies were calculated. X-ray crystal structures Human iNOS Reductase and Calmodulin Complex and Human TNF-Alpha in complex with 2-[5-(3-chloro-4-{[(1R)-1-(2-fluorophenyl)ethyl]amino} quinolin-6-yl) pyrimidin-2-yl]propan-2-ol were used from the PDB database. The docking studies were performed using the Schrödinger Glide software (Maestro 12.7) with the extra precision (XP) mode [30,31]. The best pose and binding interactions of these compounds were shown in Supplementary Figure S3. The compounds show the lowest binding energies vary from -5.0 to -8.0 shown in Supplementary Table S6. Absorption, Distribution, Metabolism and Elimination (ADME) properties of the compounds were obtained from the pharmacokinetics and pharmacodynamics parameters and assess the drug-likeness. The QikProp (Schrodinger^®^) is the prediction tool used for the evaluation of some important ADME parameters and their permissible ranges shown in Supplementary Table S7, 8.

## 4 Discussion

The main objective of this study is to screen the cardioprotective effect of GP in *in-vitro* conditions using H9c2 cells and in the animal model against ISO-induced cardiotoxicity. For the assessment of bioactivity, the antioxidant assays and *in vitro* lipid peroxidation assays were mainly considered keeping in mind the mode of action of ISO in the model. For further ascertaining if GC has any cytotoxic effects and is capable of mitigating oxidative stress, the cell- culture assays of cell viability, LDH release and intracellular ROS detection assays were performed. Taking the results obtained from these assays only, GC was then taken to animal study for further evaluation.

GP is used as a medicinal fruit, especially to treat stomach-related disorders, and is also used for culinary purposes. However, its therapeutic potential is still under-explored. Hence, this study attempted to ascertain the cardioprotective potential of GP fruit in isoproterenol-induced myocardial infarction in Wistar rats. The crude extract of GP was obtained in methanol and then fractionated. All the fractions were compared with the crude extract for comparing bioactivities in terms of antioxidant potential using radical scavenging activity (DPPH and ABTS), metal chelating assays (FRAP and PMD), and *in-vitro* lipid peroxidation inhibition potential. From the assessments, it was clear that GC exhibited the best results in antioxidant potential. Under the ferric-ascorbate-induced *in vitro* conditions in the heart tissue extracts, GC may reduce lipid peroxidation. For each of the samples taken, these activities increased dose-dependently. Also, the phenolic content was GC was found to be the highest amongst the crude and fractions of GP. These results tally with the earlier studies on multiple vegetables, spices, and medicinal herbs, where a strong positive correlation was observed between the plants’ total phenolic contents and their antioxidant potential (Aryal et al., 2019; Ulewicz-Magulska & Wesolowski, 2019; Rodríguez Madrera et al., 2021). The antioxidant capacities vary depending on the type of compounds as well; the hydrophilic antioxidants give better results in FRAP assay, and the organic ones present better results in DPPH scavenging (Ulewicz-Magulska & Wesolowski, 2019). This correlation may be attributed to the structural similarity of the phenolic compounds found in the fractions. With structural attributes like having one or more aromatic rings, hydroxyl group substitutions, and glycosylations, they are allowed to act as either electron or hydrogen donors participating in redox reactions involving free radicals, forming stable intermediates, thereby supporting their radical scavenging and metal chelating activities (Kasote et al., 2015; Singh et al., 2017). As the lipids are exposed to oxidative stress, they undergo peroxidative damage resulting in degenerative changes in the membrane fluidity and production of hydroperoxides and aldehydes like toxic by-products, which may even contribute to the pathophysiology of inflammation, liver, or cardiac complications. Moreover, it is observed that treatment with phenolic-rich plant extracts with immense antioxidant potential prevents this lipid peroxidation, as seen with the extracts of *Caralluma tuberculate* and *Annona crassiflora* (Justino et al., 2019; Poodineh & Nakhaee, 2019). Thus, the results of GC in all these assays suggest that this phenol-rich fraction has immense antioxidant potential and is carried forward for cell culture and experimental animal study to assess its cardioprotective potential.

As both MTT and LDH release assays are considered markers of cellular damage and cytotoxicity, as the former is dependent on the mitochondrial reduction of MTT into purple formazan by the live cells, while the latter is more of an indication of cellular membrane damage in apoptotic or necrotic conditions where LDH release form the damaged cells increase steeply (Aslantürk, 2018). In this study with the model of ISO-induced cellular cardiotoxicity, ISO significantly reduced cell viability by 1.5-fold while increasing the cell’s LDH release by 1.4-fold. In contrast, the pretreatment with GC for 2 h before ISO treatment successfully restricted its cardiotoxic effects, as demonstrated in significantly increased cell viability and reduced LDH release in the treated groups. Moreover, the microscopic evaluation of the morphological changes shows that GC treatment could easily protect against the cardiotoxic effects of ISO. These results align with previous publications from Cinar et al., 2021; Sivasangari et al., 2019 where the antioxidant potential of gossypin and arbutin was responsible for their cardioprotective effects. In the intracellular ROS detection assay, it was found that GC pretreatment of the cells significantly reduced ISO treatment-induced ROS generation, thereby successfully protecting the cardiac cells from oxidative stress. These results are similar to the previous reports by (Thangaiyan et al., 2018; Chen et al., 2019b; Balkrishna et al., 2021) with bioactive compounds modulating oxidative stress. From this, it can be concluded that the antioxidant potential of GC is responsible for its cardioprotective effect demonstrated in the cellular model of cardiotoxicity.

An interesting consideration here is that GC is more potent and showed better results at lower concentrations than at higher concentrations which puts forth an exciting observation that GC might act as a pro-oxidant at higher concentrations. This behavior is similar to the pro-oxidant action of vitamin C, α- tocopherol at higher concentrations (Sotler et al., 2019). In general, the antioxidant activity of the phenolic compounds is often dependent on their concentration, the pH, the presence of metal ions, and their redox potential, and changes in these may tip them off to act as pro-oxidants (Castañeda-Arriaga et al., 2018; Bayliak et al., 2016).

Isoproterenol, is a sympathomimetic *ß*- adrenoreceptor agonist which is used to induce myocardial infarction, ischemia and cardiac fibrosis like conditions in experimental models and is used in studies pertaining to cardioprtection. Wistar albino rats, injected with two consecutive dosages of 85–100 mg/kg BW of ISO, are an ideal model for studying acute myocardial infarction (Allawadhi et al., 2018). In this model, the injection of ISO is often associated with the increase in the weight of the heart, heart tail index, and heart weight index measured as a parameter for the hypertrophy of the heart (Othman et al., 2017; Hu et al., 2019). Also, the fundamental parameters used in diagnostic procedures for the detection of MI include ECG, along with pathological markers like CK- MB, CK- NAC, LDH, SGOT, SGPT, and ALP. It has been deduced that ISO-inflicted damage to the heart tissues often results in a marked increase in heart rate and a distinct ST- elevation in the ECG. It also shows a noticeable increase in all the pathological markers mentioned above. These changes indicate the presence of myocardial necrosis, damaged membrane integrity, and ischemic injury (Panda et al., 2017; Bhatt et al., 2020). With GC pretreatment, successful reduction of these hypertrophic and pathological markers down to their normal levels was recorded in this study. These results positively indicate that GC could preserve the typical membrane architecture of the myocardium, acting against the induction of cardiac hypertrophy and cardiotoxicity. These GC results align with the earlier reports of (Hu et al., 2019; Bhatt et al., 2020; Feriani et al., 2020; Soumya et al., 2021).

Moreover, apart from CK-MB and CK, cardiac troponin I (cTnI) is the most sensitive diagnostic biomarker for MI. With GC pretreatment, the reduced cardiac troponin I level indicated that the hearts were well protected against ISO-induced MI and its related damage. These findings are in agreement with the studies on the cardioprotective effect of *Fumaria indica*, bergapten, and *Curcuma longa* (Nahar and Akhter, 2018; Sajid et al., 2022; Yang et al., 2022). The increase in heart rate, prolonged QRS and QT interval, pronounced ST elevation, and shortened R-R interval and P- duration in the ECG of the ISO group reflected the presence of infraction, improper conduction of electrical impulses throughout cardiac tissues, AV node, and an overall cumulative cardiotoxic effect of isoproterenol (Boarescu et al., 2019; Bocsan et al., 2021). GC pretreated groups showed marked improvement in their ECG parameters, further providing evidence favoring its protective effect, and these detailed changes in ECG are in agreement with the earlier reports of (Jain et al., 2018; Boarescu et al., 2019; Bocsan et al., 2021; Sajid et al., 2022).

After ISO injection, the assessment of the serum lipid profile showed significant dysregulation with a notable increase in triglycerides, cholesterol, LDL-C, and VLDL-C and a steep decline in HDL-C levels. These results indicate a hyperlipidemic effect that may have occurred due to altered lipid metabolism in the presence of ISO indicating a direct correlation with myocardial infarction (Panda et al., 2017; Lekshmi & Kurup, 2019). An evident decline in triglycerides, total cholesterol, LDL-C, and VLDL-C have been observed after pretreatment with GC in this study, with a substantial increase in the HDL-C levels, revealing the hypolipidemic potency of this fraction. These results are in agreement with the studies of Panda et al., 2017; Lekshmi & Kurup, 2019; Shaik et al., 2020; Soumya et al., 2021. Moreover, from the HMG CoA reductase assay done by the indirect method where the ratio of HMG CoA/Mevalonate is calculated, and this ratio is inversely proportional to the HMG CoA reductase activity. The more the ratio, the lesser the mevalonate formed, indicating a reduction in HMG CoA reductase activity and consequently reduced cholesterol synthesis (Lekshmi & Kurup, 2019; Pramod et al., 2020). The hypolipidemic activity of GC might result from its HMG CoA reductase activity reduction, which controls cholesterol synthesis. This result is similar to the hypolipidemic and HMG CoA reductase activity reducing capability of sulphated polysaccharides from *Padina tetrastromatica* (Lekshmi & Kurup, 2019).

Excessive free radicals’ production with ISO often leads to peroxidation of membrane lipids, which might even lead to necrosis in myocardial tissues. This damage to phospholipids increases the malondialdehyde levels in the tissues, which is detected using the TBARS assay. This resembles the extensive damage to the myocardium in humans during MI. Moreover, with the increased oxidative damage and overaccumulation of peroxide and superoxide anions, compromises the functionality of the endogenous antioxidant system (SOD, GSH, and catalse), further enhancing the ROS-induced damage (Panda et al., 2017; Ahsan et al., 2022). In cardioprotective studies with sericin, bergapten, phenolic compounds rich extracts of *Zygophyllum album*, *Kedrostis foetidissima*, it was seen that their successful treatment reduced the lipid peroxidation and revamped the endogenous antioxidant system, thereby protecting the cardiac muscles against the oxidative damage. This study’s results agree with these reports from Pavithra et al., 2020; Feriani et al., 2020; Yang et al., 2022; and Ahsan et al., 2022. The role of GC pretreatment in successfully reducing TBARS in cardiac tissues and increasing the levels of SOD, GSH, and catalase proved its potent antioxidant potential which is responsible for its successful cardioprotective action.

The enhanced expression of pro-inflammatory cytokines (e.g., TNF-α, IL-6, IL-1β) and the decline in anti-inflammatory cytokine (e.g., IL- 10) establish the ISO aggravated assault on the inflammatory state in the heart tissue (Bocsan et al., 2021; Gao et al., 2021). Moreover, GC pretreated groups also showed potent anti-inflammatory activity. It reduced the pro-inflammatory cytokines TNF-α, IL-6, and IL- 1β while increasing the anti-inflammatory cytokine IL10. These results align with Adedapo et al., 2019; Kumari et al., 2020; Gao et al., 2021; Bocsan et al., 2021. NO has an intricate role in the normal smooth functioning of the cardiovascular system and is synthesized by the action of NO synthases of three isoforms, viz. endothelial or eNOS, neuronal or nNOS, and the inducible or iNOS in mammals. eNOS is responsible for NO production in normal endothelium (Ibrahim et al., 2018). A dramatic increase in serum NO and iNOS/NOS2 is evident in the ISO-treated groups, indicating the cytotoxic effects in the form of nitrosative stress, endothelial dysfunction, and increased inflammation (Hassanien, 2020). This experiment shows that GC pretreatment could neutralize the abnormal induction of iNOS, thereby reducing serum NO and tissue iNOS/NOS 2 levels, respectively. These findings are much similar to the earlier reports of Ibrahim et al., 2018; Hassanien, 2020; Khalaf et al., 2020; Sultan et al., 2022.

Even in histopathological analysis with both H&E and Masson trichome staining, the cardioprotective potency of GC was evident from how well the tissue histoarchitecture was protected in the GC—treated groups compared to ISO. These observations are similar to the earlier reports of Shang et al., 2019; Mohammed et al., 2020; Khalaf et al., 2020; Viswanadha et al., 2020.

## 5 Conclusions and future prospects

In conclusion, the findings demonstrated that GP is one of the promising dietary choices and has superior antioxidant characteristics that can be exploited to prevent cardiac hypertrophy by reducing oxidative stress and inflammation in the hypertrophic heart. The findings of the current study also suggest that GP fruit includes several phenolic antioxidants that may be responsible for the fruit’s beneficial impacts on cardiac health, including hydroxycitric acid, hydroxycitric acid lactone, parvifoliquinone, GB-1a, Garcinone A, 9-Hydroxycalabaxanthone, Chlorogenic acid, and Garcinol. However, further investigation can be done to look into GP’s potential for developing potent drugs.

## Data Availability

The original contributions presented in the study are included in the article/Supplementary Materials, further inquiries can be directed to the corresponding authors.
