# Supplementary material for: Cardioprotective potential of the antioxidant-rich bioactive fraction of Garcinia pedunculata Roxb. ex Buch.-Ham. against isoproterenol-induced myocardial infarction in Wistar rats
Source: Front Pharmacol. 2022 Oct 4;13:1009023. doi: 10.3389/fphar.2022.1009023 (PMC9577557; doi:10.3389/fphar.2022.1009023)
Supplement: Supplementary file 1 [file Table1.docx]

Supplementary Data Sheet

TABLE 1. Total phenolic content of different extracts (mg GAE/g)

| **Total Phenolic Content**  **(mg GAE/g dry extract)** | GPC | 37.16 ± 0.2886^*^ |
| --- | --- | --- |
|  | GH | 116.61 ± 3.6980^*,#^ |
|  | GC | 257.83 ± 7.5074^#^ |
|  | GE | 17.44 ± 1.3368^*,#^ |
|  | GM | 7.33 ± 1.9221^*,#^ |

(All values are as mean ± SD for triplicates. The significance has been calculated via one-way ANOVA, followed by Tukey’s post hoc test with * - p< 0.05 compared to GC, and # - p< 0.05 compared to GPC.)

TABLE 2. The radical scavenging potential of the crude extract and fractions are represented in IC_50_ values

| **Sl. No.** | **Assay** | **Samples** | **IC_50_ (µg/mL)** |
| --- | --- | --- | --- |
| 1. | DPPH Radical Scavenging Assay | Ascorbic Acid | 13.56 ± 1.2472 |
|  |  | GPC | 318.66 ± 2.1195 ^*, $^ |
|  |  | GH | 62.98 ± 1.8344 ^*,$,#^ |
|  |  | GC | 31.81 ± 1.4253 ^$,#^ |
|  |  | GE | 375.74 ± 5.3396 ^*, $,#^ |
|  |  | GM | 407.59 ± 3.8850 ^*, $,#^ |
| 2. | ABTS Radical Scavenging Assay | Ascorbic Acid | 15.81 ± 3.1364 |
|  |  | GPC | 373.78 ± 8.3045 ^*, $^ |
|  |  | GH | 51.53 ± 2.4623 ^*,$,#^ |
|  |  | GC | 36.31 ± 4.7099 ^$,#^ |
|  |  | GE | 371.91 ± 21.7984 ^*, $,#^ |
|  |  | GM | - 1. ± 5.2582 ^*, $,#^ |

(All values are in mean ± SD for triplicates. The significance has been calculated via one-way ANOVA, followed by Tukey’s post hoc test with * - p< 0.05 as compared to GC, # - p< 0.05 as compared to GPC, $ - p< 0.05 as compared to Ascorbic acid.)

TABLE 3. The reducing potential of FRAP and PMD assay of the crude extract and fractions in terms of their EC_50_ values

| Sl. No. | Assay | Samples | EC_50_ (µg/mL) |
| --- | --- | --- | --- |
|  | FRAP Assay | Ascorbic Acid | 16.19 ± 3.4346 |
|  |  | GPC | 747.63 ± 13.1015 ^*, $^ |
|  |  | GH | 414.24 ± 5.9386 ^*,$,#^ |
|  |  | GC | 216.14 ± 13.8743 ^$,#^ |
|  |  | GE | 987.04 ± 6.2015 ^*, $,#^ |
|  |  | GM | 1124.28 ± 15.1363 ^*, $,#^ |
|  | PMD Assay | Ascorbic Acid | 6.56 ± 2.8671 |
|  |  | GPC | 560.35 ± 6.7283 ^*, $^ |
|  |  | GH | 385.31 ± 3.9779 ^*,$,#^ |
|  |  | GC | 269.61 ± 10.9951 ^$,#^ |
|  |  | GE | 514.30 ± 13.2682 ^*, $,#^ |
|  |  | GM | - 1. ± 8.4890 ^*, $,#^ |

(All values are as mean ± SD for triplicates. The significance has been calculated via one-way ANOVA, followed by Tukey’s post hoc test with * - p< 0.05 as compared to GC, # - p< 0.05 as compared to GPC, $ - p< 0.05 as compared to Ascorbic acid).

TABLE 4. The comparison of the crude extract and fractions in terms of their IC_50_ values for *in vitro* lipid peroxidation assay

| Sl. No. | Assay | Samples | IC_50_ (µg/mL) |
| --- | --- | --- | --- |
| 1. | *In- vitro* lipid peroxidation assay | Trolox | 91.24 ± 3.7053 |
|  |  | GPC | 992.58 ± 9.2326 ^*, $^ |
|  |  | GH | 614.92 ± 7.3873 ^*,$,#^ |
|  |  | GC | 310.76 ± 11.6898 ^$,#^ |
|  |  | GE | 953.93 ± 16.1966 ^*, $^ |
|  |  | GM | - 1. ± 15.7394 ^*, $,#^ |

(All values are as mean ± SD for triplicates. The significance has been calculated via one-way ANOVA, followed by Tukey’s post hoc test with * - p< 0.05 as compared to GC, # - p< 0.05 as compared to GPC, $ - p< 0.05 as compared to Trolox).


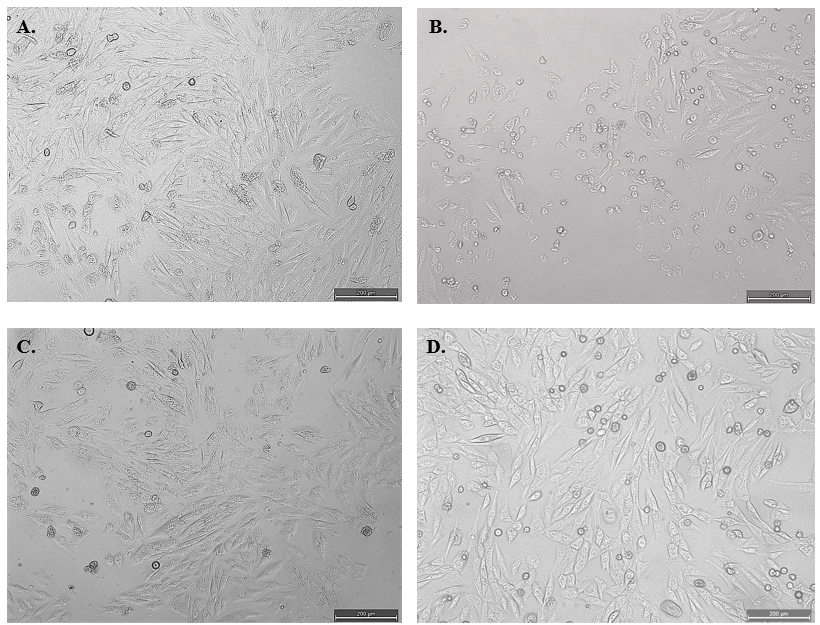


FIGURE 1. Microscopic observation of the morphological changes in H9c2 cells following ISO and GC treatments. Representative images above are of [A] Normal untreated control, [B] ISO, [C] ISO + GC (0.5 µg/mL), [D] ISO + GC (0.75 µg/mL). Scale- 200 µm.

TABLE 5. Initial and final body weight of animals in different groups during the experimental period.

| Body weight (g) | IG1 | IG2 | IG3 | IG4 | IG5 |
| --- | --- | --- | --- | --- | --- |
| Initial | 157.75 ± 6.8693 | 138.25 ± 5.494 | 114.25 ± 3.3447 | 117 ± 5.8736 | 118.25 ± 4.7631 |
| FInal | 181.9 ± 5.0906 | 167.5 ± 1.1202 | 140.9 ± 4.4637 | 142.5 ± 8.9861 | 143.4 ± 6.6588 |


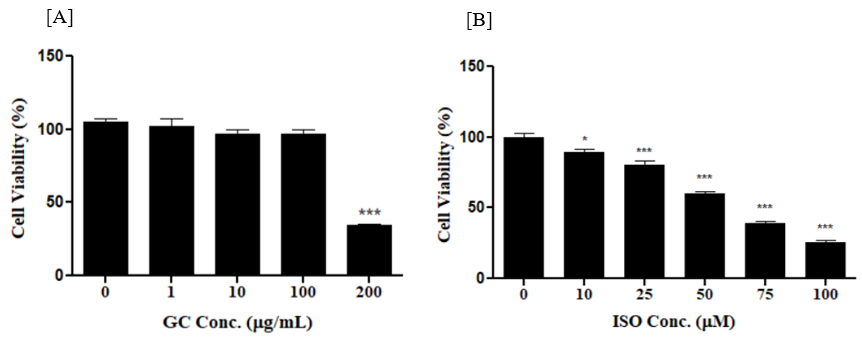


FIGURE 2. Cell viability assay (MTT) for [A] GC and [B] ISO in different concentrations (µg/mL) for toxicity study in the H9c2 cell line. Data expressed as mean ± SD for groups of three observations. *** (Statistically significant (P < 0.001) compared to untreated control group as calculated by one-way ANOVA followed by Dunnet’s multiple comparison tests).


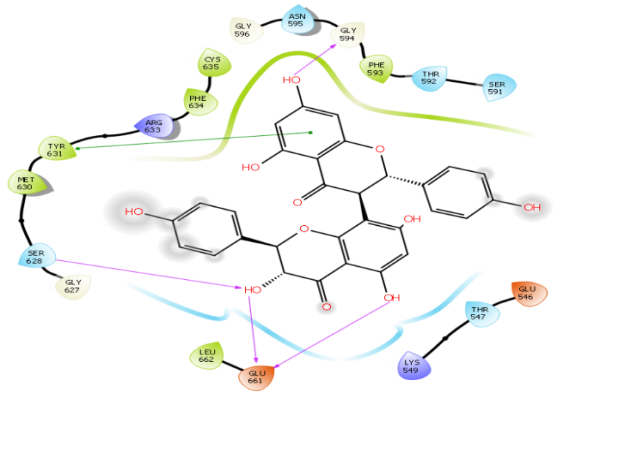

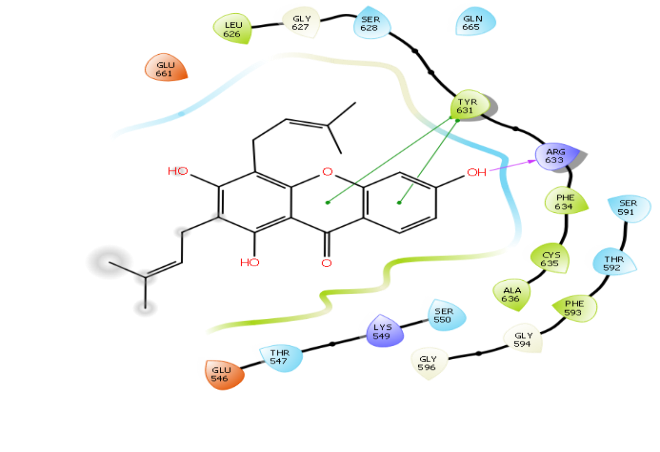

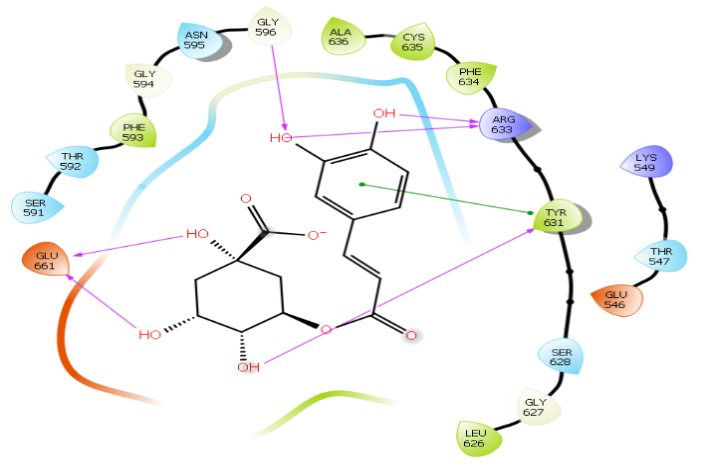

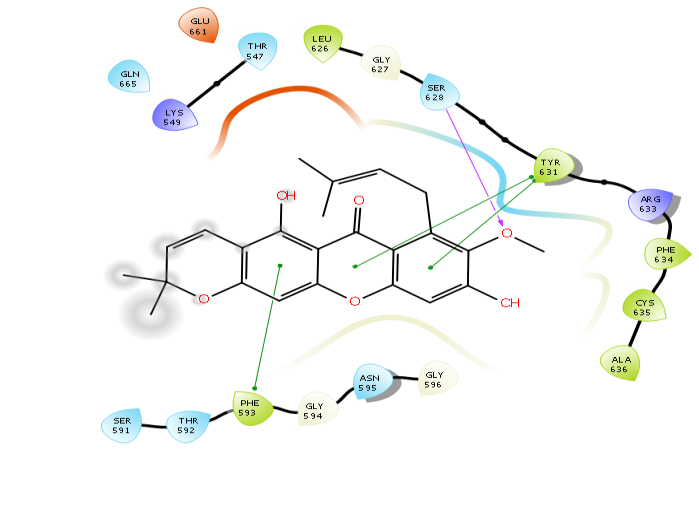

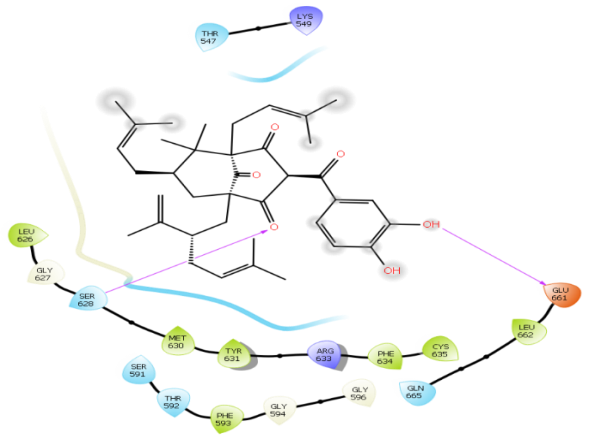

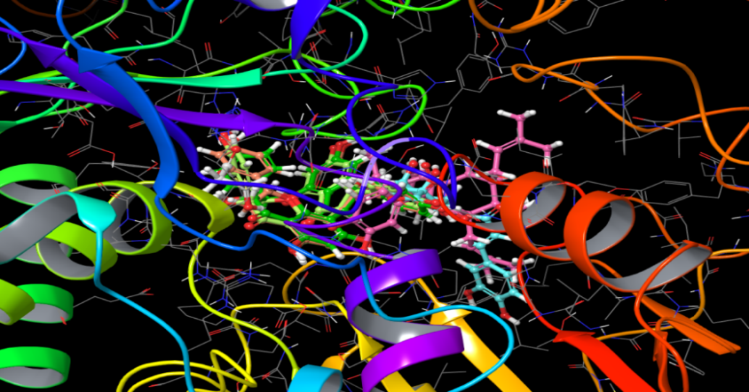


FIGURE 3. 3D & 2D Predicted binding mode of the identified compounds

TABLE 6. The lowest binding energy for the ligand

| Ligands | 3HR4: Human iNOS Reductase and Calmodulin Complex | | | | 7JRA: HUMAN TNF-ALPHA IN COMPLEX WITH 2-[5-(3-chloro-4-{[(1R)-1-(2-fluorophenyl)ethyl]amino}quinolin-6-yl)pyrimidin-2-yl]propan-2-ol | | | |
| --- | --- | --- | --- | --- | --- | --- | --- | --- |
|  | G  Score | Lipophilic  EvdW | H  Bond | Electro | G  Score | Lipophilic  EvdW | H  Bond | Electro |
| Garcinol | -5.21 | -36.86 | -1.68 | -0.39 | -7.2 | -38.66 | -1.6 | -0.41 |
| Chlorogenic acid | -6.83 | -37.61 | -1.65 | 0 | -7.3 | -39.21 | -1.6 | 0 |
| 9-Hydroxy Calabaxanthone | -6.67 | -40.56 | -1.50 | -0.43 | -7.38 | -42.66 | -1.5 | -0.42 |
| Garcinone A | -6.21 | -39.96 | -1.03 | -0.34 | -7.88 | -38.16 | -1.0 | -0.31 |
| GB-1a | -7.99 | -38.16 | -2.55 | -1.37 | -7.9 | -37.17 | -2.5 | -1.3 |
| Garcinia biflavanoid | -7.29 | -37.72 | -3.49 | -0.42 | -7.9 | -38.12 | -3.4 | -0.40 |

TABLE 7. Evaluation of drug-like properties of the identified compounds

| Molecules | mol_MW | Donor  HB | Accpt  HB | QP  polrz | QP  logPw | QP  logPo/w | QP  logS | CIQP  logS | QP  logHERG |
| --- | --- | --- | --- | --- | --- | --- | --- | --- | --- |
| Garcinol | 602.809 | 2 | 9.5 | 59.51 | 12.829 | 5.852 | -6.363 | -8.113 | -4.767 |
| Chlorogenic acid | 354.313 | 5 | 9.65 | 30.78 | 20.539 | -0.25 | -2.52 | -2.853 | -3.311 |
| 9-Hydroxy Calabaxanthone | 408.45 | 1 | 4.5 | 42.7 | 8.115 | 4.85 | -6.345 | -6.738 | -5.396 |
| Garcinone A | 380.44 | 2 | 3.75 | 41.467 | 8.749 | 4.543 | -6.605 | -6.182 | -5.754 |
| GB-1a | 542.498 | 4 | 8 | 53.51 | 19.029 | 2.873 | -6.354 | -8.441 | -6.503 |
| Garcinia biflavanoid | 558.497 | 5 | 9.7 | 51.464 | 21.948 | 1.659 | -5.292 | -8.016 | -6.19 |

TABLE 8. Calculated pharmacokinetics parameters for the test compounds

| Molecules | QP  PCaco | QP  logBB | QP  PMDCK | QP  logKp | #metab | QP  logKhsa | Human  Oral  Absorption | Percent  Human  Oral  Absorption | PSA |
| --- | --- | --- | --- | --- | --- | --- | --- | --- | --- |
| Garcinol | 530.455 | -1.518 | 249.303 | -2.388 | 14 | 1.124 | 1 | 84.06 | 110.648 |
| Chlorogenic acid | 520.768 | -3.317 | 0.666 | -6.161 | 5 | -0.926 | 1 | 100 | 185.475 |
| 9-Hydroxy Calabaxanthone | 1245.921 | -0.685 | 627.428 | -2.117 | 6 | 0.945 | 1 | 100 | 84.768 |
| Garcinone A | 322.955 | -1.536 | 145.811 | -3.135 | 9 | 0.932 | 1 | 100 | 92.304 |
| GB-1a | 4.536 | -3.412 | 1.45 | -6.081 | 10 | 0.71 | 1 | 29.604 | 190.786 |
| Garcinia biflavanoid | 2.238 | -3.689 | 0.676 | -6.668 | 10 | 0.292 | 1 | 4.041 | 209.677 |
